# Chemerin/ChemerinR1 axis and inflammation-related diseases

**DOI:** 10.3389/fmolb.2026.1785693

**Published:** 2026-04-30

**Authors:** Jun Li, Changhao Mao, Ke Li, Yan Huang, Yuhan Yang, Kunyi Li, Shuang Li, Lan Wen

**Affiliations:** 1 School of Clinical Medicine, Chengdu Medical College, Chengdu, China; 2 Department of Neurology, The First Affiliated Hospital of Chengdu Medical College, Chengdu, China; 3 Key Laboratory of Neural Injury and Repair at Chengdu Medical College of Sichuan Province, Chengdu, China; 4 Department of Neurosurgery, The First Affiliated Hospital of Chengdu Medical College, Chengdu, China

**Keywords:** Chemerin/ChemerinR1 axis, inflammation-related diseases, Chemerin peptides, G protein-coupled receptor, cardiovascular diseases, cerebrovascular diseases, metabolic diseases, immunological disease

## Abstract

Inflammation-related diseases, including cardiovascular diseases, immune disorders, and infectious diseases, have high prevalence and disability rates, imposing heavy socioeconomic and healthcare burdens. Thus, elucidating the molecular mechanisms underlying inflammation and identifying therapeutic targets are critical for the treatment of inflammation-related diseases. Chemerin, an adipokine and chemotactic protein, primarily mediates its biological effects through ChemerinR1, a G protein-coupled receptor (GPCR). Over the past 3 decades, the Chemerin/ChemerinR1 axis has been implicated in the regulation of various physiological processes, such as inflammation, metabolism, and immune response. We have comprehensively reviewed this axis plays an important role in the onset, progression, and prognosis of inflammation-related diseases, highlighting its translational potential for understanding these diseases and developing targeted therapies. This review aims to elucidate the specific mechanism of the Chemerin/ChemerinR1 axis in inflammation-related diseases. Specifically, the Chemerin/ChemerinR1 axis activates the mitogen-activated protein kinase (MAPK), PI3K/Akt, and NF-κB signaling pathways, thereby further regulating cell functions and the initiation and progression of inflammation. We also elucidate the effect of the Chemerin/ChemerinR1 axis on various inflammation-related diseases, such as hypertension, atherosclerosis, stroke, obesity, rheumatoid arthritis, and inflammatory bowel disease. Additionally, the Chemerin/ChemerinR1 axis can serve as a biomarker for various inflammation-related diseases, enabling early diagnosis, disease monitoring, and evaluation of treatment effects. Therefore, targeting the Chemerin/ChemerinR1 axis holds great potential for the treatment of vascular inflammatory diseases.

## 1 Introduction

Inflammation is a fundamental pathological mechanism implicated in a wide range of conditions including cardiovascular, immune-mediated, and infectious diseases (Liberale et al., 2022; Tang et al., 2026). These conditions represent major global health threats owing to their high prevalence, disability, and mortality. With population aging and evolving lifestyle patterns, the incidence of inflammation-related diseases is increasing, imposing substantial socioeconomic and healthcare burdens (Wu et al., 2023). Consequently, elucidating the molecular mechanisms underlying vascular inflammation and identifying novel therapeutic targets are pressing priorities in cardiovascular research (Wang et al., 2015).

Chemerin, an adipokine and chemotactic mediator, exerts its biological effects primarily through chemokine-like receptor 1 (CMKLR1, designated as ChemerinR1), a G protein-coupled receptor (GPCR). The Chemerin/ChemerinR1 axis is involved in the regulation of various physiological functions, such as metabolism, angiogenesis, anti-apoptosis and immunity. Furthermore, the Chemerin/ChemerinR1 axis plays a dual role in the inflammatory response and inflammation-related diseases (Evans et al., 2025; Wittamer et al., 2003; Bondue et al., 2011).

Previous studies have reviewed Chemerin and its receptors in metabolism and inflammation (Zhang J. et al., 2023; Stojek, 2017; Helfer and Wu, 2018). However, the function and mechanism of the Chemerin/ChemerinR1 axis during different stages of inflammation remain unclear. In the review, we systematically elaborate the molecular mechanisms of the Chemerin/ChemerinR1 axis during different stages of inflammation and its functions on inflammation-related diseases. Additionally, this review also discuss current the status of clinical research on the Chemerin/ChemerinR1 axis and future strategies for targeting the Chemerin/ChemerinR1 axis. Targeting this pathway holds considerable translational potential for developing novel diagnostic biomarkers and therapeutic strategies against inflammation-related diseases (Imiela et al., 2025; Treeck et al., 2019).

## 2 Basic functions of the Chemerin/ChemerinR1 axis

### 2.1 Origin and structure of chemerin

Chemerin, initially identified as retinoic acid receptor responder protein 2 (RARRES2) or tazarotene-induced gene 2 protein (TIG2), was first discovered in psoriatic lesions by Nagpal et al., in 1997 (Stojek, 2017). This protein is synthesized as a 163-amino acid polypeptide called pre-pro-Chemerin. Structurally, it consists of three domains: an N-terminal hydrophobic leader sequence (20 aa) essential for extracellular secretion, a central 137-amino acid region featuring a cysteine protease inhibitor-like (cystatin) fold that mediates protein–protein interactions, and a C-terminal 6-amino acid precursor fragment that serves as the primary site for proteolytic activation (Wittamer et al., 2003; Goralski et al., 2007). The pre-pro-Chemerin undergoes post-translational processing to remove the 20-residue N-terminal signal peptide, yielding the inactive precursor pro-Chemerin (Chemerin-S163) with 143 amino acids and a relative molecular mass of approximately 18 kDa (Helfer and Wu, 2018). Pro-Chemerin exhibits broad tissue expression, with the highest levels observed in the liver, white adipose tissue, lungs, and pituitary gland and moderate expression in the skin, adrenal glands, pancreas, and kidneys (Goralski et al., 2007; Segal et al., 2007). At the cellular level, pro-Chemerin mRNA is widely detected in epithelial cells, fibroblasts, endothelial cells (ECs), chondrocytes, and platelets (Banas et al., 2013).

Pro-Chemerin has no biological activity and requires protease-dependent cleavage to generate its active form at the C-terminus. This process is tightly regulated, and serine proteases play a central role therein (Cash et al., 2010). Depending on the protease involved, distinct forms of Chemerin are produced, each exhibiting differential bioactivity. For example, the low-activity Chemerin-K158 is generated by plasmin, tryptase, and factor XIa. High-activity Chemerin-S157 is produced by cathepsin K/L or elastase (Goralski et al., 2007). Moreover, high-activity Chemerin-F156 and its inactive forms (e.g., Chemerin-A155, Chemerin-F154, and Chemerin-G152) result from the cleavage of cathepsin G, chymotrypsin, and serine protease kinase-releasing enzymes (Segal et al., 2007; Wittamer et al., 2004).

**FIGURE 1:**
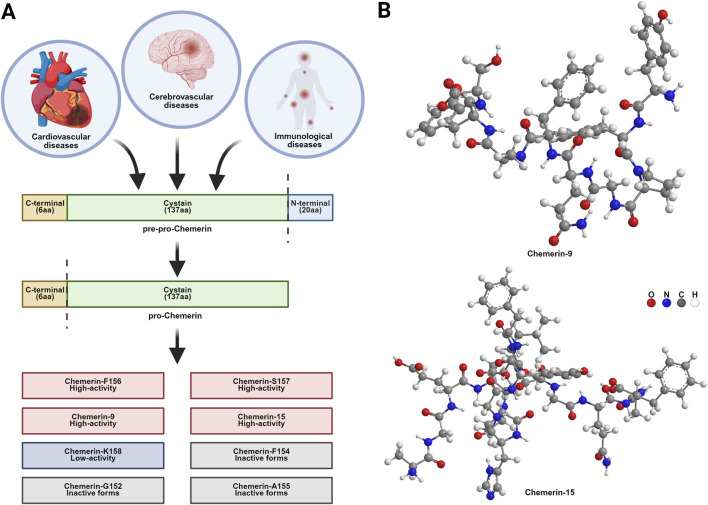
Structure of Chemerin. **(A)** Proteolytic processing of chemerin. Chemerin is produced as a pre-pro-chemerin (163aa) with no activity. The N-terminal cleavage of pre-pro-chemerin forms an inactive precursor protein, pro-chemerin (143aa). Pro-chemerin was cleaved by different protease to produce different Chemerin isoforms. **(B)** The three-dimensional structures of Chemerin-9 and Chemerin-15.

Chemerin-F154 was originally found in human hemofiltrate (Huang et al., 2020). Therein, leukocyte elastase cleaves the Ser157-Lys158 to generate Chemerin-S157, the predominant active form found in ascites (Gao et al., 2022). The human Chemerin form terminating at Lys158 (Chemerin-K158) exhibits significantly reduced potency (EC50 = 54 nM), whereas Ala155 (Chemerin-A155) is completely inactive. In contrast, the most bioactive form, Chemerin-S157, demonstrates markedly higher efficacy (EC50 = 1.2 nM) (Bondue et al., 2011). Chemerin-9 (C9; Y149-S157; YFPGQFAFS), processed human Chemerin minus six amino acids at the C-terminal (Chen et al., 2021), retains most of the activities of the Chemerin-S157 (EC50: 5 nM for C9 vs. 0.1–0.2 nM for Chemerin-S157) (Sato et al., 2019). Cash et al. identified conserved residues by aligning the sequences of putative Chemerin orthologs across several species and subsequently designed a series Chemerin-derived peptides, including C11 (P144–A154; PHGYFLPGQFA), C13 (P144–S156; PHGYFLPGQFAFS), and Chemerin-15 (C15) (A140–A154; AGEDPHGYFLPGQFA) (Cash et al., 2008). Among them, C15 has been studied in depth and has a powerful anti-inflammatory role (Cash et al., 2013) (Table 1) (Figure 1).

**TABLE 1:** The role of Chemerin and Chemerin Peptides in inflammation.

Category	Chemerin isoforms	Contained amino acid fragment	Involved signaling pathways	Exerted functions	Inflammation-related research models	References
Low-activity form	Chemerin-K158	Chemerin fragment terminating at Lys158	Low activity, weak activation of signaling pathways	Low biological activity, no obvious strong pro-inflammatory or anti-inflammatory effects	Mouse peritonitis, CHO-K1 cells and derivative cell lines model, human peripheral blood mononuclear cell model	Wittamer et al. (2004), Huang et al. (2020)
High-activity form	Chemerin-S157	Chemerin fragment terminating at Ser157	PI3K/Akt, MAPK (ERK1/2, p38), NF-κB	High biological activity; recruits monocyte migration, promotes differentiation, activates inflammatory responses; increases vascular permeability	Mouse acute peritonitis model, human umbilical vein endothelial cell (HUVEC) model, RAW264.7 mouse macrophage model	Bondue et al. (2011), Goralski et al. (2007), Segal et al. (2007), Wittamer et al. (2004), Gao et al. (2022)
High-activity form	Chemerin-F156	Chemerin fragment terminating at Phe156	PI3K/Akt, MAPK, NF-κB	Has high biological activity; participates in the initiation and amplification of inflammatory responses, and promotes inflammatory cell infiltration	Rat adjuvant-induced arthritis model, THP-1 human monocyte cell line model	Cash et al. (2008), Zabel et al. (2005)
Chemerin-derived peptide (high activity)	Chemerin-9 (C9)	Y149-S157 (sequence: YFPGQFAFS), a fragment of Chemerin-S157 with 6 C-terminal amino acids deleted	MAPK (ERK1/2, p38), PI3K/Akt, NF-κB	Retains most of the activity of Chemerin-S157; induces inflammatory responses, promotes insulin resistance; can prevent atherosclerosis	ApoE^−/−^ mouse atherosclerosis model,APP/PS1 transgenic mice model, Kunming mice model, C57BL/6 J littermate wild-type mice model, 3T3-L1 mouse adipocyte model, mouse primary microglia model, mouse primary hippocampal neurons model	Chen et al. (2021), Sato et al. (2019), Cash et al. (2008), Rennier et al. (2020), Lei et al. (2020), Zhan et al. (2025)
Chemerin-derived peptide (high activity)	Chemerin-15 (C15)	A140-A154 (sequence: AGEDPHGYFLPGQFA)	AMPK, PI3K/AKT/Nrf2, inhibits NF-κB	Potent anti-inflammatory effect; alleviates cerebral ischemia-reperfusion injury, inhibits intimal hyperplasia after angioplasty; promotes the growth and maturation of vascular endothelial cells, and reduces platelet adhesion	Rat middle cerebral artery occlusion (MCAO) model, mouse mesenteric vascular inflammation model, mouse carotid artery balloon injury model, HUVEC model	Cash et al. (2008), Huang et al. (2024), Zhang Y et al. (2023), Wen et al. (2024), Cash et al. (2013)

### 2.2 Chemerin receptors

The receptors of Chemerin include chemokine-like receptor 1, G protein-coupled receptor 1 (designated as ChemerinR2), and C–C motif chemokine-like receptor 2 (CCRL2) (Guo et al., 2012; Kennedy and Davenport, 2018). Among these, ChemerinR1 serves as the primary signaling receptor, eliciting robust intracellular signal transduction and subsequent receptor internalization upon Chemerin binding (Bondue et al., 2011). In contrast, ChemerinR2 undergoes ligand-induced endocytosis but fails to efficiently initiate classical downstream signaling cascades. CCRL2 lacks both signaling capacity and ligand-driven internalization and primarily functions as a Chemerin-presenting molecule, facilitating its bioavailability to neighboring cells (Bondue et al., 2011). The location and distribution of these receptors may help to elucidate the diversity and specificity of the biological functions of chemerins (Yoshimura and Oppenheim, 2008).

#### 2.2.1 ChemerinR1

ChemerinR1, also known as chemokine-like receptor 1, is the most extensively studied receptor with high affinity for Chemerin (Helfer and Wu, 2018; Ballet et al., 2023; Chen et al., 2024). It is a class A GPCR coupled with Gi/o that initiates intracellular calcium release, intracellular cyclic adenosine monophosphate (cAMP) reduction, and p42-p44 MAP kinase phosphorylation (Wittamer et al., 2003) (Figure 1). ChemerinR1 is highly expressed in the adipose tissue, spleen, skin, ovary, testis, and mesenteric lymph nodes (Luangsay et al., 2009), and has been detected in the prefrontal cortex, hippocampus, cerebellum, and hypothalamus of humans and rodents (Helfer et al., 2016; Peng et al., 2015). ChemerinR1 is also widely expressed in some inflammatory cells, including monocytes, macrophages, microglia, dendritic cells, NK cells, and adipocytes (Goralski et al., 2007). Recently, ChemerinR1 has been detected in human ECs and cultured human venous smooth muscle cells (Kaur et al., 2010). The biological effects of Chemerin are mainly mediated by ChemerinR1. The Chemerin/ChemerinR1 axis will be described in detail.

#### 2.2.2 ChemerinR2

ChemerinR2 is a class A GPCR that was originally cloned as an orphan receptor from human hippocampal tissue in 1994 (Kennedy and Davenport, 2018). ChemerinR2 shares significant sequence homology with ChemerinR1, although its precise physiological role remains less well-characterized than that of its paralog. In humans, ChemerinR2 mRNA was first identified in the hippocampus but not in other brain tissues (Kennedy and Davenport, 2018). High ChemerinR2 mRNA expression has been observed in the adrenal cortex, cardiomyocytes, superior cervical ganglion, and skin (Bondue et al., 2011). Additionally, ChemerinR2 mRNA and proteins are ubiquitously expressed in the smooth muscle cells of human vessels (Kennedy and Davenport, 2018; Lin et al., 2025). Human Chemerin-S157, C9, and C13 also activate ChemerinR2. Chemerin stimulation of ChemerinR2 primarily induces β-arrestin recruitment and triggers RhoA/ROCK-mediated signaling pathways (Figure 1). However, ligand-induced arrestin recruitment is not the only mode of action for ChemerinR2. This receptor exhibits constitutive internalization activity, enabling the efficient internalization of inactive peptides through an activation-independent pathway (Kennedy and Davenport, 2018).

Physiologically, ChemerinR2 has been reported in glucose homeostasis. Under high-fat diets, ChemerinR2 knockout mice show exacerbated glucose intolerance and reduced glucose-stimulated insulin secretion (Rourke et al., 2014). Chemerin also modulates steroidogenesis, as chemerin suppresses progesterone production in mouse follicles and corpus luteum via ChemerinR2. ChemerinR2 is widely expressed in human adrenal cortex, cardiomyocytes, and rodent adipose tissue, skin, and reproductive organs, supporting its roles in metabolism and reproduction (Helfer and Wu, 2018; Rourke et al., 2014).

ChemerinR2, as a co-receptor for HIV-1, HIV-2, and SIV, facilitates viral replication in brain-derived and mesangial cells (Tokizawa S et al., 2000). Additionally, its N-terminal peptide inhibits HIV infection by blocking virion binding. ChemerinR2 has been also reported in cardiovascular disease, as confirmed by regulating vascular smooth muscle cell transformation in atherosclerosis. However, lack of selective antagonists hinders further research, its dual roles in metabolism and disease highlight therapeutic potential (Kennedy and Davenport, 2018).

#### 2.2.3 CCRL2

**FIGURE 2:**
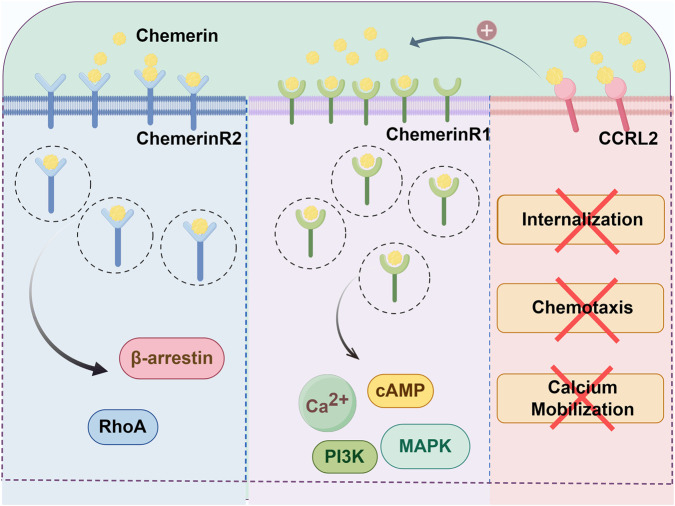
Receptors of Chemerin. The binding of Chemerin to ChemerinR1 trigger coupling with Gi/o proteins, following by intracellular calcium release, increased cyclic adenosine monophosphate (cAMP) levels, and alterations in the MAPK pathway. Chemerin binding to ChemerinR2 primarily induces β-arrestin recruitment and triggers RhoA/ROCK-mediated signaling pathways. Chemerin binding to CCRL2 does not induce CCRL2 internalization, chemotaxis, or calcium mobilization, nor trigger downstream signal transduction.

CCRL2, as a member of the chemokine receptor family, is an atypical 7-transmembrane chemokine receptor structurally similar to atypical chemokine receptors (ACKRs), but lacking chemoattractant-scavenging activity and β-arrestin activation. CCRL2 is strongly expressed by barrier cells and myeloid cells, by mast cells, at low levels by B cells, and nearly absent in other lymphocytes (including NK and T cells) (Ji et al., 2024; Annalisa Del Prete et al., 2017; Migeotte et al., 2002; Monnier et al., 2001; Lisa Patel et al., 2001). CCRL2 forms homodimers and functional heterodimers with neutrophil chemokine receptor CXCR2 at the membrane and in the cytoplasm. CCRL2 modulates CXCR2 surface expression and conformation to enhance downstream signaling (ERK1/2 phosphorylation, RhoA/Rac1 activation, Ca^2+^ mobilization, β2-integrin clustering), thereby promoting neutrophil arrest, adhesion, and extravasation at inflammatory sites (Annalisa Del Prete et al., 2017). Although it has a high affinity, similar to that of ChemerinR1 and ChemerinR2, Chemerin binding to CCRL2 fails to trigger CCRL2 internalization, chemotaxis, or calcium mobilization (Figure 2). Instead, CCRL2 serves as a non-signaling Chemerin scaffold that increases local Chemerin concentrations and binds Chemerin to its signaling receptor (e.g., ChemerinR1) in adjacent cells. This scaffold mechanism was initially proposed in early studies (Monnier et al., 2001; Zabel et al., 2008), and was recently experimentally validated as a novel regulator of NK cell lung homing (Tiberio et al., 2023).

#### 2.2.4 The interaction among ChemerinR1, ChemerinR2 and CCRL2

Chemerin binds to all three receptors but with different affinities, with high affinity for ChemerinR1 and ChemerinR2 and lower affinity for CCRL2 (De Henau et al., 2016). Functionally, ChemerinR1 is a fully functional signaling receptor mediating most chemerin-induced activities, while ChemerinR2 and CCRL2 barely activate G proteins and lack independent signaling capabilities (Yu et al., 2022) (Ben Dhaou et al., 2021). CCRL2, as an atypical non-signaling receptor, can amplify local chemerin concentration to facilitate the binding of Chemerin and ChemerinR1. Additionally, CCRL2 also presents chemerin to neighboring ChemerinR1-expressing cells and promotes ChemerinR1-mediated biological effects (Zabel et al., 2008). No direct interaction between ChemerinR1 and ChemerinR2 has been reported, but they competitively bind to chemerin due to similar affinity for the ligand (Yu et al., 2022). Collectively, ChemerinR1 is the primary functional receptor. CCRL2 acts by increasing the local concentration of chemerin and presenting it to ChemerinR1, while ChemerinR2 likely modulates ChemerinR1 activity through ligand competition (Ben Dhaou et al., 2021).

### 2.3 Activation and regulation of Chemerin/ChemerinR1 axis

As the primary signaling receptor for Chemerin, ChemerinR1 mediates most of its biological effects. We systematically reviewed the intracellular signaling cascades initiated by the Chemerin/ChemerinR1 axis and their consequent functional regulation in target cells (Treeck et al., 2019), showing that the Chemerin/ChemerinR1 signaling cascade is initiated by high-affinity ligand-receptor binding. As a member of the GPCR family, ChemerinR1 has a typical structure of seven transmembrane α-helices, and there are G protein binding sites on the C-terminal of its peptide chain and the intracellular loop connecting the fifth and sixth transmembrane helices (Kaur et al., 2010). Upon binding Chemerin to its extracellular domain, ChemerinR1 undergoes allosteric conformational changes that propagate to its intracellular regions. The third intracellular loop undergoes significant structural rearrangements, adopting a guanine nucleotide exchange factor (GEF)-like conformation that facilitates G protein activation and downstream signaling initiation (Herová et al., 2015).

The heterotrimeric G protein complex consists of three subunits: Gα, Gβ, and Gγ. In the basal state, G protein exists as a trimer (αβγ), and the α subunit binds to GDP. Upon activation by bioactive Chemerin forms (e.g., C15), ChemerinR1 undergoes conformational changes that reduce the affinity of the Gα subunit to GDP, increase its affinity for GTP, and trigger rapid GDP/GTP exchange (Cash et al., 2008). This nucleotide exchange leads to dissociation of Gα-GTP from the Gβγ dimer, exposure of previously masked effector interaction sites, and further activation various downstream signaling pathways, including the MAPK pathway, PI3K/Akt pathway, nuclear factor-κB (NF-κB) pathway and other pathways (Rennier et al., 2020).

The MAPK signaling cascade can be initiated through stimulation by either the Gα subunit or Gβγ subunit of heterotrimeric G proteins. These activated subunits trigger the conversion of Ras from an inactive GDP-bound state to an active GTP-bound conformation. As a member of the small GTPase family, Ras-GTP serves as a critical molecular switch that activates Raf kinase through direct protein–protein interactions. This initiates a sequential phosphorylation cascade: Raf phosphorylates and activates MEK, which, in turn, phosphorylates and activates ERK. Activated ERK translocates to the nucleus where it phosphorylates key transcription factors, including Elk-1 and c-Fos. These phosphorylated transcription factors then bind to specific promoter regions, modulating gene expression patterns that ultimately influence fundamental cellular processes, such as proliferation, differentiation, and survival (Cash et al., 2008). Among them, Chemerin can activate p38 MAPK through ChemerinR1 to promote the migration of endothelial progenitor cells and other cells, while inhibiting the phosphorylation of ERK1/2, thereby suppressing the proliferation of hepatocellular carcinoma cells; in contrast, its isoform C9 can activate ERK1/2 and p38 MAPK via ChemerinR1, thereby inducing inflammatory responses and insulin resistance (Lei et al., 2020; Chen et al., 2022).

The Chemerin/ChemerinR1 axis significantly modulates the PI3K/Akt signaling cascade via G protein-mediated mechanisms (Su et al., 2016). Following receptor activation, Gβγ subunits directly interact with and activate phosphoinositide 3-kinase (PI3K). This enzyme catalyzes the conversion of phosphatidylinositol-4,5-bisphosphate (PIP2) to phosphatidylinositol-3,4,5-trisphosphate (PIP3), a potent secondary messenger that accumulates in the plasma membrane. Newly generated PIP3 recruits Akt (protein kinase B) from the cytoplasm to the membrane. Subsequently, Akt is completely activated through phosphorylation modification under the action of a series of auxiliary proteins. Activated Akt phosphorylates and activates downstream proteins such as mTOR, a serine/threonine protein kinase that regulates cell growth and metabolic activities, and maintains cell survival and homeostasis (Lu et al., 2022). In this signaling pathway, Chemerin can inhibit the phosphorylation of Akt at the Ser473 site via the ChemerinR1-PTEN axis, thereby blocking the downstream signaling of the PI3K/Akt pathway and suppressing cell migration and invasion; among its isoforms (Rennier et al., 2020). C9 exerts bidirectional regulation on the PI3K/Akt pathway via ChemerinR1 in different cell types: it activates the PI3K/Akt pathway and promotes the nuclear translocation of NF-κB in some cell types (e.g., endothelial cells), while in insulin-sensitive cells (e.g., skeletal muscle cells), it inhibits insulin-induced Akt phosphorylation, ultimately exacerbating insulin resistance (Rennier et al., 2020; Zhan et al., 2025).

The nuclear factor-κB (NF-κB) pathway serves as a central regulator of inflammatory responses and immune function. In the basal state, NF-κB dimers (typically p50/p65) are sequestered in the cytoplasm through interaction with inhibitory IκB proteins. C15 binding to ChemerinR1 activates the IκB kinase (IKK) complex that phosphorylates IκBα at specific N-terminal residues (Ser32/Ser36), triggering its polyubiquitination and subsequent proteasomal degradation. This post-translational modification releases NF-κB from its cytoplasmic tether, allowing its nuclear translocation (Dranse et al., 2016). Within the nucleus, NF-κB binds to κB enhancer elements in promoter regions of target genes, inducing transcription of pro-inflammatory cytokines (e.g., TNF-α, IL-6), chemokines, and adhesion molecules. This coordinated gene expression program regulates critical biological processes, including immune cell activation, inflammatory responses, and cellular survival (Lin et al., 2017). In this pathway, Chemerin exerts a bidirectional regulatory effect: it can exert an anti-inflammatory effect by inhibiting the nuclear translocation of the p65 subunit, likely the effect exerted by its isoform C15, and can also activate NF-κB through the reactive oxygen species (ROS)-silent information regulator 1 (Sirt1) pathway to promote the inflammatory response (Jaworek et al., 2019); in contrast, its form C9 activates NF-κB via the synergistic effect of PI3K/Akt and p38 MAPK, thereby upregulating the expression of adhesion molecules such as intercellular adhesion molecule-1 (ICAM-1) and vascular cell adhesion molecule-1 (VCAM-1) (Wang et al., 2019).

In addition to classical signaling pathways, the Chemerin/ChemerinR1 axis modulates cellular functions through alternative mechanisms. The axis regulates intracellular calcium dynamics. Chemerin binding to ChemerinR1 triggers calcium mobilization from endoplasmic reticulum stores via phospholipase C (PLC)-dependent generation of inositol trisphosphate (IP3), resulting in transient cytosolic calcium elevation (Ferland et al., 2017). This calcium flux serves as a versatile secondary messenger that coordinates diverse cellular processes, including contractile activity in vascular smooth muscle cells, secretory responses in immune cells, and the metabolic reprogramming of adipocytes. Moreover, the Chemerin/ChemerinR1 axis interacts with other cell surface receptors or signaling molecules to form a complex signaling network that jointly regulates vascular inflammation. For instance, it can form heterodimers with other GPCRs, alter the functional characteristics and signal transduction patterns of the receptors, or interact with some non-GPCR signaling molecules, such as receptor tyrosine kinases, and affect intracellular signaling pathways through crosstalk. ChemerinR1 forms heterodimers with CXCR4. This interaction was detected in recombinant cell models using Bioluminescence Resonance Energy Transfer and Homogeneous Time-Resolved Fluorescence technologies (de Poorter et al., 2013). Chemerin exerts distinct functions through its binding to various receptors (Figure 2).

## 3 Relationship between the Chemerin/ChemerinR1 axis and onset and development of inflammation-related diseases

### 3.1 Inflammation-related diseases

Inflammation is a fundamental defensive response of vascularized tissues to various injurious stimuli, including biological pathogens (bacteria, viruses, and parasites), physical trauma (mechanical injury, burns, and radiation), chemical irritants (corrosive substances and toxins), and necrotic tissue components (Kim and Lee, 2024). This complex pathophysiological process is initiated by characteristic vascular changes including vasodilation and increased blood flow, which manifest clinically as localized redness and warmth. Concurrently, enhanced vascular permeability facilitates the exudation of plasma proteins and leukocytes into the interstitial spaces, resulting in tissue swelling. Subsequently, inflammatory cells are recruited to lesions and perform crucial phagocytic functions to eliminate pathogens and cellular debris while releasing a cascade of chemical mediators including cytokines (e.g., IL-1β, TNF-α), chemokines, and prostaglandins (Mariani and Roncucci, 2015). These mediators amplify the inflammatory response and stimulate nociceptors, causing pain and functional impairment associated with inflammation. The precise interplay among vascular changes, cellular infiltration, and molecular signaling pathways isolates and neutralizes harmful stimuli during tissue preparation for subsequent repair processes (Zabel et al., 2005).

The inflammatory response is involved in the onset and progression of various diseases in multiple organ systems. In infectious diseases, such as bacterial pneumonia, COVID-19, and candidiasis, inflammatory responses mediate protective immunity and pathological tissue damage (Sulicka-Grodzicka et al., 2022). Similarly, dysregulated inflammation drives the development and progression of autoimmune disorders including rheumatoid arthritis, psoriasis, systemic lupus erythematosus, asthma, and inflammatory bowel diseases (ulcerative colitis and Crohn’s disease) (Berg et al., 2010). Even in mechanical injuries such as fractures, localized inflammatory cascades initiate the healing process.

Vascular pathologies demonstrate the dual nature of inflammation: acute responses promote tissue repair, whereas chronic inflammation contributes to pathological processes such as post-angioplasty intimal hyperplasia and atherosclerotic plaque formation through endothelial dysfunction and lipid accumulation. Inflammation-associated chronic diseases include hypertension (McMillan and Kirabo, 2025), diabetic vasculopathy, Alzheimer’s disease, chronic obstructive pulmonary disease (COPD), obesity-related metabolic dysfunction, chronic cerebral hypoperfusion, ischemic stroke, and persistent hepatitis. These conditions share common inflammatory mechanisms characterized by sustained immune cell infiltration, persistent cytokine release, and maladaptive tissue remodeling, which collectively drive disease progression (Luangsay et al., 2009). The ubiquitous involvement of inflammatory pathways in diverse pathologies highlights their central role in both physiological defense and pathological processes.

### 3.2 Chemerin/ChemerinR1 axis in the initiation of inflammation

During the initiation phase of inflammation, the Chemerin/ChemerinR1 axis plays a critical role in two core pathological events: (1) inducing or inhibiting vascular endothelial dysfunction, and (2) mediating the early recruitment of inflammatory cells. Vascular ECs are essential as the primary barrier to the vascular wall for maintaining vascular homeostasis. Under physiological conditions, ECs sustain vasodilation, inhibit platelet aggregation and leukocyte adhesion, and ensure normal vascular function by secreting bioactive substances such as nitric oxide (NO) and prostacyclin (PGI2) (Bondue et al., 2011). However, exposure to pathological stimuli (e.g., oxidative stress, hyperlipidemia, and hypertension) disrupts this balance and triggers endothelial dysfunction.

In the inflammatory state, Chemerin inhibits the activity of endothelial nitric oxide synthase (eNOS) and reduces nitric oxide production, further impairing vascular diastolic function, increasing the risk of platelet aggregation and leukocyte adhesion, and promoting vascular inflammation (Lei et al., 2020). *In vitro* experiments show that ECs treated with Chemerin exhibit downregulated expression of intercellular junction proteins (e.g., occludin and claudin) (Shang et al., 2019), leading to widened gaps between ECs and increased permeability. This facilitates the entry of lipids and inflammatory cells from the bloodstream into the subendothelial space, thereby driving the onset of atherosclerosis.

Among various chemokines and cytokines released by activated ECs, Chemerin is a key regulator of inflammation. Its production marks the critical transition from endothelial dysfunction to active vascular inflammation. Secreted Chemerin rapidly binds to ChemerinR1 on the surface of vascular endothelial cells and circulating immune cells, initiating a series of inflammatory cascades (Bondue et al., 2011; Cash et al., 2010).

In the early stages of atherosclerosis, Chemerin released by vascular ECs forms a chemotactic signal via a concentration gradient, recruiting monocytes to migrate to the lesion sites (Tang et al., 2023). This chemoattractant signaling ensures the precise targeting of endothelial injury sites and serves as an initial link for inflammatory cell infiltration. Migrating monocytes undergo phenotypic transformation into macrophages in the subintimal space. Experimental evidence from animal models of atherosclerosis has demonstrated that exogenous Chemerin administration significantly enhances macrophage accumulation beneath the vascular intima, highlighting the central role of the Chemerin/ChemerinR1 axis in monocyte/macrophage trafficking (Bondue et al., 2011).

At the molecular level, the primary mediator of these effects is the active full-length Chemerin, generated by the protease-mediated hydrolysis of pro-Chemerin rather than the C15 fragment (Yoshimura and Oppenheim, 2008). In the early stages of inflammation, epithelial cells, adipocytes, and other cell types secrete inactive pro-Chemerin, which is hydrolyzed into active full-length Chemerin by enzymes such as cathepsin L and trypsin (Kulig et al., 2011). This active form binds with a high affinity to ChemerinR1 on the surface of immune cells (neutrophils and monocytes), triggering chemotactic recruitment signals. Upon binding, it activates the PI3K-Akt and MAPK pathways (ERK1/2 and p38) (Kaneko et al., 2011).

The PI3K-Akt pathway promotes actin cytoskeleton rearrangement in immune cells, enhancing their migratory capacity, and guiding neutrophils and monocytes to accumulate at inflammatory sites. The MAPK pathway activates downstream transcription factors (e.g., AP-1), inducing the expression of adhesion molecules (e.g., ICAM-1) (Kim et al., 2021; Xie and Liu, 2022). This finding further supports the idea of immune cell adhesion to the vascular endothelium and transendothelial migration, ultimately initiating an inflammatory response (Cash et al., 2010).

**FIGURE 3:**
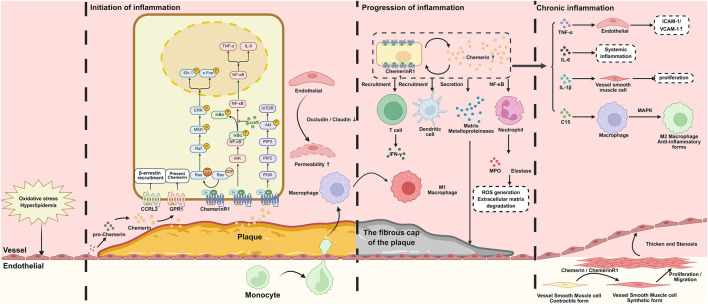
Schematic diagram of Chemerin/ChemerinR1 axis in different stages of inflammation. In the initial stage of inflammation, pathological stimuli (e.g. oxidative stress, hyperlipidemia) impair EC function. Chemerin binding to ChemerinR1 on ECs reduces tight junction proteins (e.g. occludin, claudin) and promote inffammatory cells enter the subendothelial space. Chemerin-S157 released from activated ECs binds to ChemerinR1 on the monocyte/macrophages, and activates PI3K/Akt/MAPK pathways. In the progressing stage of inflammation, macrophages phagocytose ox-LDL to become foam cells. The foam cells secrete Chemerin and upregulate ChemerinR1, forming a cycle to recruit neutrophils, T lymphocytes, dendritic cells. Additionally, activated macrophages, neutrophils and T lymphocytes release TNF-α, IL-6, and MMPs via NF-κB pathway. These inflammatory cytokines and cells contribute to chronic inflammation. ECs, endothelial cells; ox-LDL, oxidized low density lipoprotein; MMPs, matrix metalloproteinases.

In addition to monocyte and macrophage recruitment, the Chemerin/ChemerinR1 axis also attracts other immune cell populations (e.g., dendritic cells and neutrophils) to migrate to vascular inflammation sites. Chemerin binding to ChemerinR1 significantly enhances the chemotactic responsiveness of dendritic cells and neutrophils during the initial inflammatory phase (Wittamer et al., 2003). Although the number of these infiltrating cells is relatively small in the early stage, they achieve “directional navigation” to the inflammatory site via the Chemerin/ChemerinR1 axis—laying the groundwork for massive accumulation in the subsequent inflammatory progression phase (Parolini et al., 2007). Collectively, these mechanisms constitute the fundamental initiating events of inflammatory cascades, underscoring the important role of this chemokine axis in vascular pathobiology (Cash et al., 2008) (Figure 3).

### 3.3 Impact of the Chemerin/ChemerinR1 axis on inflammation progression

After vascular inflammation progresses from the initiation stage to the progression stage, the Chemerin/ChemerinR1 axis drives the progressive exacerbation of the inflammatory response through multiple mechanisms, including establishing a positive feedback regulatory network, expanding the range of inflammatory cell infiltration, promoting the release of inflammatory mediators, and impairing vascular structural integrity. This accelerates the progression of vascular lesions and promotes vascular inflammation to more complex pathological stages (Eichelmann et al., 2019).

A core feature of the inflammatory progression stage is “excessive recruitment” of inflammatory cells, driven by positive feedback regulation of the Chemerin/ChemerinR1 axis. Residual and recruited cells at injured lesions continuously secrete Chemerin and upregulate ChemerinR1 expression on their surface, forming a “secretion–binding–re-secretion” positive feedback loop (Helfer and Wu, 2018). This loop increases Chemerin concentration at the inflammatory site, strengthens chemotactic signals, and attracts more types of immune cells (e.g., T-lymphocytes, neutrophils, and dendritic cells) to accumulate in the vascular wall. Their massive infiltration spreads the inflammatory response from local to broader vascular areas (Ge et al., 2018).

The Chemerin/ChemerinR1 axis exerts precise regulatory effects on different cell types. Chemerin binds to ChemerinR1 on its surface to guide chemotaxis to the inflammatory site, with active full-length Chemerin as the primary mediator. This not only sustains activation of the PI3K-Akt and MAPK pathways but also additionally activates the NF-κB pathway via the ChemerinR1 axis (Dimitriadis et al., 2018). NF-κB activation promotes neutrophils to express and release large amounts of inflammatory mediators and proteases (e.g., myeloperoxidase [MPO] and elastase). MPO catalyzes the production of substantial amounts of ROS, causing oxidative stress damage, further disrupting vascular endothelial integrity, directly degrading extracellular matrix components, loosening the vascular wall structure, and creating conditions for deeper inflammatory cell infiltration (Rosales et al., 2016). Excessive neutrophil activation triggers the release of neutrophil extracellular traps (NETs). While NETs capture pathogens in normal immunity, their excessive release in the progressive stages of vascular inflammation directly damages vascular ECs and induces platelet aggregation (Thiam et al., 2020).

The activation and proliferation of T-lymphocytes are pivotal for inflammatory progression. In the vascular inflammatory microenvironment, the interaction between Chemerin and ChemerinR1 on the surface of T-lymphocytes directly promotes their activation and proliferation. Furthermore, the expression level of ChemerinR1 in T-lymphocytes within atherosclerotic plaques is positively correlated with Chemerin concentration (Albanesi et al., 2009), indicating that the binding affinity between Chemerin and ChemerinR1 directly influences T-lymphocyte activity. Activated T-lymphocytes secrete various pro-inflammatory cytokines, including interferon-γ (IFN-γ) and interleukin-2 (IL-2). IFN-γ further activates macrophages, enhancing their phagocytic capacity and secretion of pro-inflammatory factors to amplify the inflammatory response (Lensing and Jabbari, 2022); IL-2 maintains the stability of inflammatory cell populations by promoting T-lymphocyte proliferation and differentiation, ensuring the persistence of the inflammatory response. Furthermore, among T-lymphocyte subsets, helper T cells 1 (Th1) secrete increased levels of pro-inflammatory factors (e.g., IFN-γ) under Chemerin modulation, while the anti-inflammatory function of regulatory T cells (Treg) is suppressed (Helfer and Wu, 2018). This Th1/Treg cell imbalance further aggravates inflammation progression. This axis also meticulously regulates the polarization of various immune cells, thereby influencing the progression and outcomes of inflammation.

Dendritic cells, recognized for their potent antigen-presenting capabilities, are also involved in inflammatory progression, a process regulated by the Chemerin/ChemerinR1 axis. This axis significantly enhances the antigen-presenting function of dendritic cells by activating the NF-κB signaling pathway, thereby strengthening their ability to stimulate T-lymphocytes (Su et al., 2021). Consequently, activated T-lymphocytes secrete pro-inflammatory cytokines (e.g., IFN-γ and TNF-α), forming an amplification cascade involving “dendritic cells–T cells–inflammatory factors” and advancing the inflammatory response to a more severe stage.

In terms of inflammatory mediators, after binding to ChemerinR1, full-length Chemerin activates the NF-κB pathway to regulate the transcriptional balance between pro-inflammatory cytokines (TNF-α, IL-6, and IL-1β) and anti-inflammatory cytokines (IL-10) (Zabel et al., 2006; Brian et al., 2005). This ensures the release of pro-inflammatory factors to clear pathogens, while promoting the release of IL-10 to prevent excessive inflammatory amplification. For example, in atherosclerosis-related inflammation, the sustained action of full-length Chemerin promotes continuous TNF-α release, which induces vascular ECs to express more adhesion molecules (e.g., ICAM-1, VCAM-1), enhancing leukocyte-endothelial adhesion and migration and further aggravating the inflammatory response (Özcan et al., 2015).

In contrast, C15, a C-terminal 15-peptide fragment of Chemerin, exerts anti-inflammatory effects (Cash et al., 2014). C15 binds to ChemerinR1 and inhibits NF-κB activation by enhancing the stability of IκB, which prevents NF-κB from translocating into the nucleus, thereby suppressing the transcription of pro-inflammatory factors. In the inflammatory microenvironment, C15 can directly downregulate the expression of TNF-α and IL-6 in macrophages and dendritic cells while upregulating the release of the anti-inflammatory factor IL-10 (Huang et al., 2024).

The Chemerin/ChemerinR1 axis also regulates matrix metalloproteinases (MMPs). Full-length Chemerin promotes the synthesis and release of various MMPs (e.g., MMP-1, MMP-2, and MMP-9) (Berg et al., 2010). These enzymes extensively degrade extracellular matrix components (e.g., collagen and elastin), causing significant damage to the structural integrity of the vascular wall. In atherosclerotic plaques, excessive MMP activity gradually thins the fibrous cap on the plaque surface, thereby significantly reducing plaque stability and increasing the risk of rupture (Johnson, 2017). When unstable plaques rupture, the subendothelial collagen and tissue factors are exposed, rapidly triggering platelet aggregation and thrombosis. In the coronary arteries, this can directly lead to serious cardiovascular events (e.g., acute myocardial infarction), one of the most dangerous pathological outcomes of the inflammatory progression stage (Yukihiko Momiyama et al., 2014) (Figure 3).

### 3.4 Chemerin/ChemerinR1 axis in the chronic inflammation stage

As vascular inflammation progresses to the chronic phase, the functional role of the Chemerin/ChemerinR1 axis shifts from its initial pro-inflammatory role to mediating vascular remodeling and tissue repair. However, under conditions of dysregulated immune response, sustained activation of this axis contributes to maladaptive vascular changes. Persistent inflammatory signaling, chronic immune cell activation, and pathological vascular smooth muscle cell (VSMC) proliferation collectively drive structural alterations in the vessel wall, including fibrosis, neointimal formation, and extracellular matrix deposition (Sun et al., 2021). These irreversible modifications not only perpetuate chronic vascular dysfunction but also serve as critical pathological substrates for the long-term progression of cardiovascular diseases, reinforcing the pivotal role of Chemerin/ChemerinR1 signaling in chronic vascular pathogenesis (Imiela et al., 2025).

A significant feature of the chronic stage is the “continuous action” of inflammatory factors. Under regulation of the Chemerin/ChemerinR1 axis, inflammatory factors persist for long periods and cause sustained damage to vascular tissues. For instance, in COPD, which is associated with chronic vascular inflammation, tumor necrosis factor-α (TNF-α) remains highly expressed (Arndt, 2025). Full-length Chemerin contributes to this sustained high-level TNF-α expression by activating the NF-κB pathway. TNF-α induces vascular ECs to express more adhesion molecules (e.g., intercellular adhesion molecule-1 [ICAM-1], vascular cell adhesion molecule-1 [VCAM-1]), enhancing leukocyte-endothelial adhesion and increasing the number of leukocytes migrating to the vascular wall (Fabienne Mackay et al., 1993). This forms a vicious cycle of “inflammatory cell infiltration–endothelial damage–more adhesion molecule expression,” further impairing endothelial barrier function.

Interleukin-1β (IL-1β) primarily targets VSMCs. Full-length Chemerin promotes the activation of IL-1β-related signaling pathways in VSMCs, significantly enhancing their proliferative and migratory capacities (Kunimoto et al., 2015). VSMCs migrate from the vascular media to the intima and proliferate extensively, leading to increased vascular wall thickness, gradual lumen narrowing, and severe impairment of vasodilatory and contractile functions.

Interleukin-6 (IL-6) exerts systemic effects. Full-length Chemerin promotes the activation and proliferation of T-lymphocytes by activating relevant pathways, and the activated T-lymphocytes secrete IL-6 (Lu et al., 2024). IL-6 systemically extends local inflammatory responses by regulating the synthesis of acute-phase proteins (e.g., C-reactive protein) in the liver, promoting T-lymphocyte activation and proliferation, sustaining immune cells in a persistently activated state, and rendering chronic inflammation difficult to resolve (He et al., 2015).

“Continuous activation” of inflammatory cells is another core feature of chronic inflammation, with macrophages playing a key role. Under the regulation of the Chemerin/ChemerinR1 axis, macrophages in the chronic phase continuously secrete Chemerin and exhibit high ChemerinR1 expression, ensuring the sustained presence of chemotactic signals at the inflammatory site and facilitating the continuous infiltration of new immune cells. In the chronic inflammatory microenvironment, full-length Chemerin promotes macrophage polarization primarily into the pro-inflammatory M1 phenotype (van der Vorst et al., 2019). M1 macrophages continuously amplify the inflammatory response by secreting large amounts of pro-inflammatory cytokines (e.g., TNF-α, IL-1β, IL-6), and their lipid-phagocytic function remains active—continuously promoting the formation and accumulation of foam cells and providing a material basis for the enlargement of atherosclerotic plaques (He et al., 2015; Serhan, 2014). Although C15 can regulate macrophage polarization toward the anti-inflammatory M2 phenotype, this regulation is insufficient to offset pro-inflammatory effects in the overall pro-inflammatory environment in the chronic stage, leading to persistent inflammation (Krautbauer et al., 2013). C15 binds to ChemerinR1 and activates the AMPK pathway. AMPK downregulates the activity of inflammation-related signaling molecules (e.g., mTOR) in macrophages, decreasing pro-inflammatory factor synthesis, promoting fatty acid oxidation, and supplying energy for the anti-inflammatory functions of M2 macrophages. However, the effect of C15 is limited to the strong pro-inflammatory background in the chronic stage (Serhan, 2007).

The proliferation and migration of VSMCs are the core pathological processes of vascular remodeling in the chronic stage and are regulated by the Chemerin/ChemerinR1 axis. Under normal physiological conditions, VSMCs exhibit a contractile phenotype that maintains vascular tension and structural stability (Bennett et al., 2016; Chen et al., 2023). However, under chronic inflammatory stimulation, VSMCs undergo phenotypic transformation from contractile to synthetic (Bennett et al., 2016). Synthetic VSMCs possess enhanced proliferative and migratory capacities and can synthesize large quantities of extracellular matrix components (e.g., collagen and elastin) (Ma et al., 2025). Full-length Chemerin promotes the phenotypic transformation of VSMCs by activating the PI3K-Akt and MAPK pathways and drives VSMC proliferation via the activation of related signaling pathways (Li et al., 2021). Chemerin and its different active forms exert distinct effects at various stages of inflammation (Figure 3).

Chronic stage: Full-length Chemerin binds VSMC ChemerinR1, activating PI3K/Akt/MAPK to switch VSMCs from contractile to synthetic phenotype, enhancing proliferation/migration and causing vascular wall thickening/lumen stenosis. Synthetic VSMCs secrete matrix to form plaque fibrous caps, but sustained Chemerin/ChemerinR1 activation thins caps, increasing plaque instability.

### 3.5 Dual roles of Chemerin/ChemerinR1 axis

The Chemerin/ChemerinR1 axis exhibits both anti-inflammatory and pro-inflammatory effects in inflammation. What determines whether chemerin acts in a pro- or anti-inflammatory manner remains an important question? Elucidating the regulatory factors is crucial for understanding the mechanistic role of the Chemerin/ChemerinR1 axis in modulating inflammation-related diseases and optimizing targeted therapeutic strategies. Chemerin isoform is the determining factor. Chemerin exists in multiple isoforms with distinct bioactivities, which determines the dual role of Chemerin in different inflammation-related diseases. Chemerin isoforms (e.g., Chemerin-S157, Chemerin-F156, C9) mainly exhibit pro-inflammatory state and have high affinity for ChemerinR1. Chemerin-S157 (EC50 = 1.2 nM) is highly expressed in the early stage of atherosclerosis and rheumatoid arthritis, and drives inflammatory cell recruitment and activation. Chemerin isoforms (e.g., C15, C9) shows the anti-inflammatory effect in in-stent restenosis, peritonitis, ischemic stroke and myocardial ischemia-reperfusion (Cash et al., 2013). The balance between pro-inflammatory and anti-inflammatory isoforms is regulated by protease networks. The serine proteases (cathepsin K/L, elastase) promote the generation of pro-inflammatory isoforms, while carboxypeptidases (e.g., carboxypeptidase N) mediate the cleavage of pro-inflammatory isoforms into inactive or anti-inflammatory derivatives (Wittamer et al., 2003; Cash et al., 2014). Additionally, different disease-specific microenvironments are also a critical factor. This content will be elaborated on in the following section.

## 4 Chemerin/ChemerinR1 axis and inflammation-related diseases

### 4.1 Cardiovascular diseases

#### 4.1.1 Hypertension

The Chemerin/ChemerinR1 axis plays a critical role in the initiation and progression of hypertension (Ferland et al., 2020). Abnormal contractile function of VSMCs is a key driver of elevated blood pressure in the pathogenesis of hypertension. When Chemerin binds to ChemerinR1, it activates the downstream phospholipase C (PLC)-inositol triphosphate (IP3)-calcium signaling pathway, activated PLC hydrolyzes phosphatidylinositol bisphosphate (PIP2) into IP3 and diacylglycerol; IP3 then binds to IP3 receptors on the endoplasmic reticulum, inducing calcium release and a subsequent rise in intracellular calcium concentration. Calcium ions bind to calmodulin (CaM) to activate myosin light chain kinase, which catalyzes myosin light chain. This process enhances myosin-actin interactions, triggers VSMC contraction, increases vascular resistance, and ultimately elevates blood pressure (Stojek, 2017).

Patients with hypertension often exhibit marked vascular inflammatory responses and the Chemerin/ChemerinR1 axis promotes inflammatory cell infiltration in this context. Chemerin specifically binds to ChemerinR1 on the surface of immune cells, recruiting macrophages, T-lymphocytes, and other inflammatory cells to infiltrate vascular walls. Under hypertensive conditions, damaged vascular ECs secrete Chemerin, while activated macrophages release pro-inflammatory cytokines (e.g., tumor necrosis factor-α [TNF-α], interleukin-6 [IL-6]) to further exacerbate vascular inflammation (Dranse et al., 2016). Among these mediators, TNF-α induces ECs to upregulate the expression of intercellular adhesion molecule-1 (ICAM-1) and vascular cell adhesion molecule-1 (VCAM-1), enhancing the adhesion of inflammatory cells to ECs and facilitating greater infiltration into the vascular wall.

Additionally, the Chemerin/ChemerinR1 axis modulates VSMC proliferation and migration, contributing to vascular remodeling and impairing vascular function. During the progression of hypertension, abnormal activation of this axis drives VSMCs to switch from a contractile to a synthetic phenotype. Synthetic VSMCs exhibit strong proliferative and migratory capacities and secrete extracellular matrix components (e.g., collagen and fibronectin), leading to vascular wall thickening and lumen stenosis (Man et al., 2023).

Animal experiments support these findings; injecting Chemerin into hypertensive model rats induces a rapid increase in blood pressure and a notable increase in vascular resistance (Watts et al., 2013). In contrast, the administration of ChemR23 antagonists significantly reduced the magnitude of blood pressure elevation and lowered vascular resistance in rats. These results confirm that Chemerin activates ChemR23, triggering abnormal VSMC contraction and disrupting normal blood pressure regulation, thus playing a key role in the onset and progression of hypertension (Watts et al., 2018). Studies in hypertensive mouse models have shown that inhibition of the Chemerin/ChemR23 axis reduces VSMC proliferation and migration, decreases vascular wall thickness, and improves vascular remodeling (Zhang J. et al., 2023; Kunimoto et al., 2015; Neves et al., 2015).

Therefore, the Chemerin/ChemerinR1 axis exacerbates hypertension pathogenesis by regulating VSMC contraction, promoting vascular inflammation, and influencing vascular remodeling. Future research targeting this axis is expected to yield more effective therapies for hypertension.

#### 4.1.2 Atherosclerosis

The Chemerin/ChemerinR1 axis influences atherosclerosis through multiple mechanisms. In the early stages of atherosclerosis, vascular ECs, stimulated by risk factors such as oxidized low-density lipoprotein (ox-LDL), hypertension, and hyperglycemia, upregulate Chemerin expression (Liu et al., 2019). Furthermore, various disease-related mechanisms impair the vascular endothelium. Hypertension increases intravascular pressure, enhances blood flow shear force on the arterial endothelium, and triggers damage. Unstable blood flow at vascular bifurcations and curvatures also induces mechanical injury to the endothelium (Hu et al., 2020). Under hyperglycemic conditions, biological macromolecules undergo glycosylation to form advanced glycosylation end products, which bind to receptors on vascular ECs, promoting inflammatory cytokine secretion and endothelial damage (Nam et al., 2016). Moreover, factors such as smoking, hyperlipidemia, hyperhomocysteinemia, and infection directly or indirectly damage vascular ECs, weaken the endothelial barrier, and increase vascular permeability (Aviv and Levy, 2012).

In high-fat diet-induced atherosclerotic mouse models, Chemerin mRNA and protein expression in vascular ECs are significantly upregulated, with a corresponding increase in circulating Chemerin levels (Liu et al., 2019). Concurrently, in the early stages of atherosclerosis, blood immune cells (e.g., macrophages and dendritic cells) begin to express ChemerinR1, with levels gradually increasing with disease progression. After vascular endothelial damage, blood lipid components (e.g., low-density lipoprotein [LDL], particularly ox-LDL) can enter the subendothelial space through the impaired endothelium. As atherosclerosis advances, Chemerin and ChemerinR1 expression in atherosclerotic plaques further increases, and their expression in foam cells, which are formed by macrophages phagocytosing ox-LDL, is significantly higher than that in normal macrophages (Christos G Kostopoulos et al., 2014). This is attributed to ox-LDL stimulating macrophages to synthesize and secrete more Chemerin and upregulate ChemerinR1 expression.

Macrophages phagocytose ox-LDL upon recognition, and excessive phagocytosis transforms them into foam cells. The continuous accumulation of these foam cells forms early lipid streaks that trigger an inflammatory response, which causes blood inflammatory cells (e.g., monocytes, T-lymphocytes) to accumulate at the lesion site and release inflammatory cytokines (e.g., TNF-α, interleukin-1 [IL-1]) (Fernández-Friera et al., 2019). These cytokines further damage the vascular endothelium and promote the migration of smooth muscle cells from the media to the subendothelium. During atherosclerosis, VSMCs undergo phenotypic transformation from contractile to synthetic, and synthetic VSMCs express higher levels of ChemerinR1. The binding of Chemerin to ChemerinR1 promotes VSMC proliferation and migration, leading to vascular wall thickening (Wang et al., 2015). Subsequently, smooth muscle cells synthesize and secrete extracellular matrix, which, together with deposited lipids and inflammatory cells, forms atherosclerotic plaques (Hansson and Hermansson, 2011).

In the advanced stages of atherosclerosis, even in unstable plaques, Chemerin and ChemerinR1 remain highly expressed (Serhan, 2014; Buechler et al., 2019). Chemerin binds to ChemerinR1 to activate inflammatory cells and promotes the release of inflammatory cytokines and matrix metalloproteinases (MMPs). This leads to thinning of the fibrous cap of the plaque and increases the risk of plaque rupture. Furthermore, Chemerin and ChemerinR1 expression in unstable plaques is positively correlated with MMP expression.

The Chemerin/ChemerinR1 axis also affects atherosclerosis progression through other pathways: (1) Chemerin recruits macrophages, dendritic cells, and other inflammatory cells to accumulate in the vascular wall, activates the PI3K/Akt and ERK1/2 signaling pathways, and promotes monocyte adhesion and migration to the subendothelial space (where monocytes differentiate into macrophages and further form foam cells) (Jiang et al., 2017; Mitsis et al., 2024); (2) Chemerin expression in adipocytes and the liver is closely linked to lipid metabolism, and its upregulation disrupts lipid homeostasis in these tissues, promoting lipid deposition in the vascular wall (Helfer and Wu, 2018; Tan et al., 2023).

Clinical studies have confirmed a close association between the Chemerin/ChemerinR1 axis and atherosclerosis risk and severity: plasma Chemerin levels in patients with coronary heart disease are significantly higher than those in healthy controls and are positively correlated with the degree of coronary artery stenosis (Lei et al., 2020). This promotes VSMC proliferation and migration, leading to neointimal formation and vascular stenosis. Meanwhile, activation of the Chemerin/ChemerinR1 axis stimulates the secretion of inflammatory cytokines (e.g., TNF-α, IL-6), further exacerbating vascular wall inflammation, promoting atherosclerotic lesion progression and instability, and increasing the risk of cardiovascular events (Arita et al., 2007).

### 4.2 Cerebrovascular diseases

#### 4.2.1 Stroke

Taking myocardial ischemia–reperfusion injury as an example, the C15/ChemerinR1 axis, a subaxis of the broader Chemerin/ChemerinR1 system composed of the derived peptide C15 and its receptor ChemerinR1, exerts a protective role (Lu et al., 2022). During myocardial ischemia–reperfusion, this axis mitigates the inflammatory response by regulating macrophage polarization and shifting macrophages toward the anti-inflammatory tissue-repairing M2 phenotype (Voet et al., 2019). Additionally, C15 inhibits the release of inflammatory cytokines, reduces myocardial cell damage, and promotes myocardial repair, all of which facilitate recovery of myocardial function (Liu et al., 2018).

C15 promotes the phenotypic transformation of microglia from the pro-inflammatory M1 phenotype to the anti-inflammatory M2 phenotype in cerebral ischemia models (He et al., 2015). After C15 binds to ChemerinR1, it induces receptor internalization, activates AMPK phosphorylation (p-AMPKα Thr172), inhibits NF-κB p65 nuclear translocation, and suppresses neuronal apoptosis by regulating microglial phenotypic transformation, thereby alleviating ischemia–reperfusion injury (Huang et al., 2024).

In the pathological process of ischemic stroke, the C15/ChemerinR1 axis exerts substantial neuroprotective effects (Parolini et al., 2007; Liu et al., 2021) through mechanisms including inhibition of the inflammatory response, reduction of oxidative stress damage, and promotion of neuronal survival and repair (Lu et al., 2022). First, the C15/ChemerinR1 axis reduces the production and release of inflammatory cytokines (e.g., IL-1β, IL-6, TNF-α) by inhibiting the activation of inflammatory signaling pathways (e.g., NF-κB), thereby minimizing inflammatory damage to neurons (Abareshi et al., 2021). Typical pathological changes in ischemic stroke models include neuronal pyroptosis (a form of programmed necrosis) and the exacerbation of inflammation. Neuronal pyroptosis causes substantial neuronal death and worsens brain damage, whereas a heightened inflammatory response triggers inflammatory cell infiltration and inflammatory cytokine release, forming a vicious cycle that increases the cerebral infarction volume and causes more severe neurological deficits. ChemerinR1 deletion exacerbates neuronal pyroptosis and inflammation (Zabel et al., 2005; Voet et al., 2019), whereas activating ChemerinR1 (e.g., using agonists such as RvE1 or C-9) significantly alleviates these pathological changes (Suzu et al., 2021; Hamlett et al., 2020; Rey et al., 2016). Activating this axis substantially reduces inflammatory cytokine levels and inflammatory cell infiltration in the brain tissue, and ChemerinR1 expression is upregulated after model establishment (Arita et al., 2007).

Second, this axis upregulates the expression of antioxidant enzymes (e.g., superoxide dismutase [SOD] and glutathione peroxidase [GSH-Px]) by activating antioxidant signaling pathways. This reduces ROS accumulation, protects neurons from oxidative stress damage, and regulates the intracellular redox status to maintain redox balance.

Finally, the C15/ChemerinR1 axis regulates macrophage polarization from the pro-inflammatory M1 phenotype to the anti-inflammatory M2 phenotype by activating AMPK phosphorylation (p-AMPKα Thr172) and inhibiting NF-κB p65 nuclear translocation. This suppresses neuronal apoptosis, exerts neuroprotective effects, and regulates the expression of genes associated with nerve cell regeneration and axonal growth. Activating this axis significantly improves neurological deficits (Sun et al., 2020; Arita et al., 2005), suggesting that the C15/ChemerinR1 axis protects neurons during ischemic stroke pathogenesis and may serve as a potential target for ischemic stroke treatment (Sato et al., 2019; Ben Dhaou et al., 2022; Yang et al., 2022).

#### 4.2.2 Chronic cerebral hypoperfusion (CCH)

The Chemerin/ChemerinR1 axis plays a key regulatory role in the pathological process of CCH. CCH downregulates ChemerinR1 expression in a potentially adaptive response. However, this downregulation exacerbates nerve damage and cognitive impairment (Zabel et al., 2005; Poh et al., 2022; Lee et al., 2021). Activation of ChemerinR1 induces the PI3K/AKT/Nrf2 signaling pathway to exert neuroprotective effects, inhibits NLRP3 inflammasome activation, and reduces inflammatory cytokine release and neuronal pyroptosis, thereby significantly improving CCH-induced pathological changes (Zhang Y. et al., 2023; Wang et al., 2019; Sun et al., 2020).

The specific mechanism is as follows. After PI3K activation, ChemerinR1 catalyzes PIP2 to generate phosphatidylinositol triphosphate (PIP3), which recruits and activates AKT to the cell membrane. Activated AKT then phosphorylates Nrf2 (Sun et al., 2020); phosphorylated Nrf2 enters the nucleus, binds to the antioxidant response element, and initiates the transcription of antioxidant and anti-inflammatory genes (e.g., heme oxygenase-1 [HO-1] and SOD) (Sigfridsson et al., 2020). Carbon monoxide (CO) produced by HO-1 exerts vasodilatory, anti-inflammatory, and antioxidant effects, whereas SOD reduces oxidative stress damage (Matsuyama et al., 2020).

Additionally, ChemerinR1 activation inhibits NLRP3 inflammasome-mediated neuronal pyroptosis (Liu et al., 2024). In CCH, oxidative stress and inflammatory responses activate the NLRP3 inflammasome, leading to Caspase-1 activation; this in turn cleaves IL-1β and IL-18 and induces cell pyroptosis (Lin et al., 2020; Lei et al., 2018). ChemerinR1 inhibits NLRP3 inflammasome activation via the PI3K/AKT/Nrf2 signaling pathway, reducing inflammatory cytokine release and neuronal pyroptosis (Biasizzo and Kopitar-Jerala, 2020).

In CCH mouse models, treatment with ChemerinR1 agonists (e.g., RvE1 and C-9) significantly reduces neuronal damage and improves cognitive function. These results indicate that the Chemerin/ChemerinR1 axis may have regulatory and protective effects against CCH-related nerve damage and cognitive impairment, providing new targets for the treatment of related diseases (Wang et al., 2019; Kantarci et al., 2018; Zhang et al., 2019).

### 4.3 Metabolic diseases

In patients with obesity and metabolic syndrome, expression of the Chemerin/ChemerinR1 axis is significantly increased and strongly associated with disease onset and progression (Buechler et al., 2019). Adipocyte accumulation in patients with obesity enhances Chemerin secretion, increasing serum Chemerin levels, a change that is positively correlated with body fat percentage (Du et al., 2009; Bauer et al., 2012). Patients with metabolic syndrome often have multiple metabolic disorders (e.g., hypertension, hyperglycemia, and dyslipidemia) that further stimulate cellular Chemerin secretion and lead to elevated serum Chemerin levels (Dranse et al., 2016; Bauer et al., 2012).

The Chemerin/ChemerinR1 axis contributes to the pathogenesis of obesity and metabolic syndrome through multiple mechanisms, with regulation of adipocyte function being a key link (Serhan, 2014). In adipocytes, binding of Chemerin to ChemerinR1 activates signaling pathways, inhibits fatty acid oxidation, and promotes fatty acid synthesis and storage, leading to increased fat accumulation. Meanwhile, Chemerin regulates the secretion of cytokines (e.g., TNF-α, IL-6) by adipocytes, exacerbating adipose tissue inflammation (Zhang Y. et al., 2023; Bauer et al., 2011).

Exacerbated inflammation is another important mechanism; Chemerin recruits immune cells to infiltrate adipose tissue, activates macrophages, and stimulates the secretion of inflammatory cytokines. In the adipose tissue of obese mice, macrophage infiltration increases, Chemerin and ChemerinR1 expression are upregulated, and inflammatory cytokine secretion is significantly elevated. This inflammatory response further damages adipocytes and vascular ECs, increases insulin resistance, and worsens metabolic disorders (Yang et al., 2017).

Insulin resistance, a key pathological feature of obesity and metabolic syndrome, is strongly influenced by the Chemerin/ChemerinR1 axis. Chemerin inhibits insulin signal transduction in adipocytes and hepatocytes, directly increasing insulin resistance and exerting indirect effects by regulating inflammatory cytokine secretion. Elevated insulin resistance further increases blood glucose levels, worsens metabolic disorders, and creates a vicious cycle that accelerates the progression of obesity and metabolic syndrome (Bondue et al., 2011; Xie and Liu, 2022; Zhang et al., 2018).

### 4.4 Immunological diseases

#### 4.4.1 Rheumatoid arthritis (RA)

RA is a chronic inflammatory joint disease driven by complex immune responses and inflammatory processes. The Chemerin/ChemerinR1 axis plays an important role in RA, with significantly elevated Chemerin expression strongly associated with disease activity and inflammatory markers (Berg et al., 2010; Kim et al., 2020; Carrión et al., 2019). Chemerin promotes the secretion of inflammatory cytokines by activating the NF-κB pathway. After Chemerin binds to ChemerinR1 on the surface of synoviocytes and macrophages, it activates IκB kinase (IKK), leading to IκB degradation. NF-κB then enters the nucleus and promotes the transcription and secretion of inflammatory cytokines (e.g., TNF-α, IL-1β, IL-6). These cytokines not only promote synoviocyte activation, induce chondrocytes and osteocytes to produce MMPs, and cause joint tissue damage, but also activate T and B cells, further exacerbating inflammation (Berg et al., 2010; Kaneko et al., 2011).

The axis also affects synoviocyte proliferation and migration through ChemerinR1-mediated signaling pathways and binds to ChemerinR1 on the surface of synoviocytes, activating pathways such as ERK1/2, p38 MAPK, and PI3K/Akt (Kaneko et al., 2011; Carrión et al., 2019). Among these, activation of ERK1/2 promotes the expression of cyclin D1 and cyclin-dependent kinase 4 (CDK4), driving cells from the G1 phase to the S phase and promoting synoviocyte proliferation. Furthermore, activation of p38 MAPK enhances synoviocyte migratory capacity by regulating cytoskeletal rearrangement (Hsu et al., 2006). Activation of the PI3K/Akt pathway causes Akt to phosphorylate downstream substrates (e.g., mammalian target of rapamycin [mTOR]), activating proteins such as p70S6K and 4E-BP1 downstream of mTOR, promoting protein synthesis, and thereby stimulating synoviocyte proliferation. Excessive synoviocyte proliferation and migration lead to synovial hyperplasia and synovial tissue thickening, further exacerbating joint damage (Carrión et al., 2019).

The Chemerin/ChemerinR1 axis also plays a critical role in intra-articular angiogenesis. Chemerin promotes vascular endothelial cell proliferation, migration, and tube formation, thereby facilitating intra-articular angiogenesis. Angiogenesis is significantly increased in the synovial tissue of patients, and newly formed blood vessels provide channels for inflammatory cell infiltration and inflammatory factor transport, further driving inflammation progression. Chemerin binds to ChemerinR1 on the surface of ECs to activate pathways such as PI3K/Akt and MAPK, thereby promoting the expression and secretion of angiogenesis-related factors, such as vascular endothelial growth factor [VEGF] (Kiymet Bozaoglu et al., 2010). VEGF binds to its receptors on vascular ECs, activates downstream signaling pathways, and stimulates endothelial cell proliferation and migration to form new blood vessels. This axis regulates extracellular matrix degradation and remodeling, creating a favorable microenvironment for angiogenesis.

In RA mouse models, inhibition of the Chemerin/ChemerinR1 axis activity significantly reduces intra-articular angiogenesis, alleviating joint inflammation and damage, and improving joint function (Carrión et al., 2019; Chen et al., 2013). This indicates that the Chemerin/ChemerinR1 axis is a potential target for RA treatment and that specific antagonists or neutralizing antibodies can be developed to modulate this axis for RA management.

#### 4.4.2 Inflammatory bowel disease (IBD)

IBDs, such as ulcerative colitis (UC) and Crohn’s disease (CD), are chronic inflammatory intestinal disorders in which intestinal vascular inflammation drives disease progression. In patients with IBD, the Chemerin/ChemerinR1 axis is dysregulated, with markedly elevated Chemerin levels in intestinal tissue and serum, and changes intricately linked to disease onset and progression (Gunawan et al., 2022). Chemerin levels are notably higher in CD patients than in healthy individuals; in CD patients, Chemerin mRNA and protein expression in intestinal mucosal tissue are significantly upregulated, and serum Chemerin levels are positively correlated with the Crohn’s Disease Activity Index (DAI). A similar trend is observed in UC patients, where increased Chemerin levels in intestinal tissue and serum are associated with disease severity. These findings suggest that Chemerin plays a crucial role in the pathogenesis of IBD (Lin et al., 2014).

The Chemerin/ChemerinR1 axis participates in the pathological process of IBD through multiple mechanisms, with regulation of intestinal immune cell recruitment being the key. Chemerin binds to ChemerinR1 on the surface of immune cells, recruiting inflammatory cells (e.g., macrophages, dendritic cells, and T-lymphocytes) to accumulate at intestinal inflammatory sites. Its binding to macrophages activates the PI3K/Akt and ERK1/2 signaling pathways, promoting macrophage migration and activation, followed by the secretion of inflammatory cytokines (e.g., TNF-α, IL-1β, IL-6) that exacerbate inflammation (Song et al., 2022). The recruited dendritic cells, which act as antigen-presenting cells, activate T-lymphocytes to trigger specific immune responses, leading to persistent inflammatory aggravation (Vermi et al., 2005).

Activation of the axis promotes the secretion of inflammatory cytokines by intestinal epithelial cells. For instance, under the Chemerin/ChemerinR1 axis stimulation, intestinal epithelial cells secrete IL-8 and chemokine ligand 2 (CCL2): IL-8 attracts neutrophils and intensifies inflammation, whereas CCL2 recruits monocytes to promote inflammatory cell infiltration. In IBD mouse models, blocking this axis significantly reduces the secretion of inflammatory cytokines by intestinal epithelial cells and effectively alleviates intestinal inflammation (Zhao et al., 2014; Waluga et al., 2014).

The regulation of inflammatory cytokines is another important mechanism. After Chemerin binds to ChemerinR1, it activates pathways such as NF-κB and MAPK, promoting the expression and release of inflammatory cytokines (Yang et al., 2022). In the intestinal tissue of IBD patients, these pathways are activated, and the expression of inflammatory cytokines (e.g., TNF-α, IL-1β, IL-6) is significantly increased. These cytokines damage intestinal epithelial cells, impair intestinal mucosal barrier function, increase intestinal permeability, and allow harmful substances (e.g., bacteria and endotoxins) to enter intestinal tissues, further exacerbating inflammation. Additionally, Chemerin affects the recruitment and activation of inflammatory cells by regulating the expression of other inflammatory mediators (e.g., chemokines and adhesion molecules) (Lin et al., 2014; Song et al., 2022; Waluga et al., 2014).

Furthermore, patients with IBD often exhibit extra-intestinal vascular inflammation, and the Chemerin/ChemerinR1 axis plays an important role in this process. The increased risk of cardiovascular disease in patients with IBD may be associated with extra-intestinal vascular inflammation. Elevated Chemerin reaches the vascular system through the bloodstream, binds to ChemerinR1 on the surface of vascular endothelial and immune cells, and triggers vascular inflammatory responses. In vascular ECs, this binding activates pathways such as NF-κB and MAPK, increasing the expression of inflammatory cytokines (e.g., TNF-α, IL-6). These cytokines damage vascular ECs, leading to endothelial dysfunction, reduced nitric oxide (NO) secretion, and increased endothelin-1 (ET-1) production. This results in vasoconstriction and elevated blood pressure, while also promoting the adhesion and infiltration of inflammatory cells to exacerbate vascular inflammation. This axis may affect lipid deposition in the vascular wall by regulating lipid metabolism, further promoting the onset and progression of extra-intestinal vascular inflammation (Imiela et al., 2025; Weigert et al., 2010).

### 4.5 Other inflammation-related diseases

C15 exerts a notable inhibitory effect on intimal hyperplasia after angioplasty. After angioplasty (e.g., vascular stent implantation), intimal hyperplasia is a major pathological mechanism leading to in-stent restenosis, which is associated with inflammatory responses and the proliferation and migration of VSMCs. In post-angioplasty intimal hyperplasia, C15 alleviates the postoperative inflammatory response through its anti-inflammatory properties, reduces the release of inflammatory cytokines, and inhibits monocyte adhesion (Jourd’heuil et al., 2016). It also induces the transformation of smooth muscle cells (SMCs) from a synthetic to a contractile phenotype, reducing their proliferative and migratory capabilities, thereby inhibiting excessive intimal hyperplasia.

Additionally, stents immobilized with C15 promote the growth and maturation of ECs, and reduce inflammatory cell infiltration (Wen et al., 2024; Li et al., 2021). C15 also regulates the polarization of macrophages toward the anti-inflammatory M2 phenotype, reducing the secretion of pro-inflammatory cytokines. In the Chandler loop experiment (simulating blood flow), stents immobilized with C15 significantly reduced platelet adhesion and activation, and decreased the formation of platelet-leukocyte complexes, thus inhibiting the inflammatory response.

In *in vivo* experiments, C15-immobilized stents were implanted into the iliac arteries of rabbits. The stents significantly reduced excessive neointimal hyperplasia and decreased the incidence of vascular restenosis. In addition, C15 stents accelerated endothelialization, promoted the formation of a complete and mature intima, and reduced inflammatory cell infiltration (Wen et al., 2024).

The Chemerin/ChemerinR1 axis plays an important role in various vascular inflammatory diseases through shared mechanisms and effects. Chemerin expression is significantly increased in vasculitis (e.g., granulomatosis with polyangiitis and microscopic polyangiitis) (Imiela et al., 2025; Abu-Shakra et al., 2021). Chemerin binds to ChemerinR1, recruits and activates T-lymphocytes and macrophages, releases inflammatory mediators, and causes vascular wall inflammation. The infiltration of ChemerinR1-positive cells into lesion tissues is strongly associated with Chemerin expression levels and disease activity, indicating that this axis is involved in the pathogenesis of vasculitis (De Palma et al., 2011).

In COVID-19 patients (involving pulmonary and systemic vascular inflammation), Chemerin expression shows dynamic changes; it is significantly reduced in patients with moderate and severe disease and then increases during the 28-day follow-up period, suggesting that Chemerin may be involved in inflammation resolution. Additionally, Chemerin is negatively correlated with the levels of inflammatory markers (e.g., TNF-α, IL-1β, PTX3), implying its potential role in inflammation resolution. In pulmonary and systemic vascular inflammation caused by COVID-19, the Chemerin/ChemerinR1 axis may affect disease progression and outcomes by regulating the inflammatory response (Sulicka-Grodzicka et al., 2022).

In summary, the Chemerin/ChemerinR1 axis regulates inflammatory responses, activates inflammatory signaling pathways, promotes the secretion of cytokines and metalloproteinases, recruits and activates immune cells, and causes endothelial cell damage and vascular wall inflammation in diseases, such as atherosclerosis, acute myocardial infarction, ischemic stroke, vasculitis, and diabetic vascular disease (Zabel et al., 2008).

## 5 Clinical transformation prospects

### 5.1 Chemerin/ChemerinR1 axis as a biomarker

The Chemerin/ChemerinR1 axis exhibits distinct changes in various vascular inflammatory diseases, making it a potential biomarker for disease diagnosis and monitoring. In atherosclerosis, serum Chemerin levels correlate positively with coronary artery stenosis severity, plasma Chemerin levels are significantly higher in patients with coronary heart disease than in healthy controls, and plasma Chemerin levels correlate positively with carotid intima-media thickness (IMT) in patients with carotid atherosclerosis, supporting its utility in assessing disease progression (Lei et al., 2020; He et al., 2015). Chemerin levels are significantly higher in patients with acute coronary syndrome than in patients with stable angina pectoris and healthy controls; therefore, Chemerin levels can be used to predict the degree of myocardial injury (Cash et al., 2010; He et al., 2015). In patients with RA and ankylosing spondylitis, Chemerin expression is associated with disease activity and inflammatory markers.

In patients with hypertension, serum Chemerin levels are closely related to blood pressure control, which is significantly higher than that in healthy controls, and positively correlated with systolic and diastolic blood pressure. Furthermore, Chemerin level is positively correlated with carotid IMT in patients with hypertension, making it a potential indicator of the degree of vascular lesions. In patients with diabetic vascular disease, serum Chemerin levels in patients with type 2 diabetes are not only positively correlated with carotid IMT but also associated with the severity of lower extremity vascular disease and urinary albumin excretion rate (Imiela et al., 2025; Gu et al., 2015). Therefore, detecting the expression of Chemerin or ChemerinR1 in the blood or tissues facilitates the early detection of vascular inflammatory diseases, evaluation of disease severity and progression, and provides a critical basis for clinical treatment decisions.

### 5.2 Chemerin/ChemerinR1 axis as a therapeutic target

Interventions targeting the Chemerin/ChemerinR1 axis are effective in treating vascular inflammatory diseases. Inhibiting the activity of this axis, for example, using ChemerinR1 antagonists or knocking out ChemerinR1 via gene editing technology, can reduce the recruitment and activation of inflammatory cells, decrease the secretion of inflammatory cytokines, alleviate vascular inflammatory responses, and inhibit the proliferation and migration of VSMCs, thus preventing and managing diseases such as atherosclerosis and post-angioplasty intimal hyperplasia (Lei et al., 2020; He et al., 2015).

Conversely, activation of the Chemerin/ChemerinR1 axis also has therapeutic potential in certain cases. For example, in ischemic stroke and CCH, ChemerinR1 agonists (e.g., RvE1 and C-9) can activate related protective signaling pathways, reduce neuronal damage, and improve neurological function (Banas et al., 2013; Sato et al., 2019; Dranse et al., 2016).

Chemerin-neutralizing antibodies are another potential drug intervention strategy. They specifically bind to Chemerin, block its interaction with ChemerinR1, and inhibit signal transduction (Xu et al., 2019). In studies of diabetic vascular disease, Chemerin-neutralizing antibodies reduce damage to vascular ECs, decrease the expression of inflammatory cytokines, and inhibit platelet aggregation and thrombosis. Additionally, these antibodies improve insulin resistance and reduce blood glucose levels (Xie and Liu, 2022). Therefore, precise regulation of the activity of the Chemerin/ChemerinR1 axis based on the pathological characteristics of different diseases provides a new direction for personalized treatment.

### 5.3 Drug development

Drug development based on the Chemerin/ChemerinR1 axis is ongoing. Current research directions include the design of small-molecule inhibitors and antibodies to specifically block the ChemerinR1 signaling pathway and reduce inflammatory responses using gene editing technology (e.g., CRISPR-Cas9) to knock out the expression of Chemerin or ChemerinR1, thus intervening in the function of this axis at the genetic level, which provides an innovative method to treat vascular inflammation (Stojek, 2017; Özcan et al., 2015; Liu et al., 2024).

Additionally, some natural compounds (e.g., berberine) regulate the Chemerin/ChemerinR1 axis and are expected to be further developed and optimized for clinical treatment in the future (Ye et al., 2015). The application of vascular stents loaded with anti-inflammatory C15 peptides via nanotechnology to reduce intimal hyperplasia and promote vascular healing also provides additional research and clinical application opportunities for the derived C15 peptide.

### 5.4 Current status and clinical research challenges

Clinical research on the Chemerin/ChemerinR1 axis is still in its early stages, focusing on the detection of related indicators and exploration of their association with diseases. Small-scale clinical studies have confirmed that changes in the expression of this axis in diseases, such as atherosclerosis, hypertension, and diabetic vascular disease, are strongly associated with disease severity and prognosis (Imiela et al., 2025). However, most of these studies are cross-sectional, lacking long-term follow-up and intervention studies, and there is limited in-depth understanding of the dynamic changes of this axis during disease development and the effectiveness and safety of intervention measures.

During clinical transformation, multiple technical challenges exist: (1) In detecting Chemerin and ChemerinR1 expression, there is a lack of standardized methods and reliable reagents; differences in methods and reagents may lead to inconsistent results, affecting the accuracy and comparability of studies. (2) During drug development, it is necessary to address the issues of drug targeting and delivery to ensure that drugs act accurately on this axis while avoiding the effects on normal cells and physiological processes. Commonly used drug delivery systems (e.g., liposomes and nanoparticles) require optimization in terms of stability, targeting, and biocompatibility.

Safety is a key issue in clinical transformation. Interventional measures may interfere with the body’s immune regulatory function, thereby increasing the risk of infection or causing immune disorders. Although gene therapy has potential, it carries safety hazards, such as off-target effects, immunogenicity, and carcinogenic risks. Therefore, sufficient safety evaluations and long-term follow-up studies are required prior to clinical application.

In terms of effectiveness, although animal experiments have shown efficacy, human physiological and pathological processes are more complex, and drug metabolism and mechanisms of action may differ from those in animal models. Therefore, large-scale clinical trials are needed to verify the effectiveness and safety of intervention measures in humans while also considering the impact of individual differences on treatment outcomes.

## 6 Conclusion

The Chemerin/ChemerinR1 axis plays a crucial role in the development of vascular inflammation. In terms of basic characteristics, Chemerin is generated by the cleavage of inactive precursors by proteases, yielding multiple forms with distinct biological activities. ChemerinR1, its receptor, is widely distributed in immune cells, vascular ECs, VSMCs, and adipocytes, providing a basis for axis functions. After activation, the axis regulates cell biological behavior through a series of complex signal transduction processes, including cAMP inhibition and the MAPK, PI3K/Akt, and NF-κB pathways (Conde et al., 2011).

In terms of its mechanism of action in vascular inflammation, this axis significantly regulates the recruitment and activation of inflammatory cells, endothelial cell function, the proliferation and migration of SMCs, and inflammatory signal transduction and cytokine secretion. Specifically, it guides inflammatory cells to migrate to vascular inflammatory sites, regulates macrophage polarization, and affects neutrophil chemotaxis and phagocytosis; regulates the proliferation, migration, and apoptosis of ECs, promoting endothelial repair under physiological conditions but potentially causing endothelial dysfunction under pathological conditions; promotes the transformation of SMCs from a contractile to a synthetic phenotype; and activates inflammatory signaling pathways such as NF-κB and MAPK, promoting the secretion of inflammatory cytokines and MMPs and amplifying the inflammatory response.

The abnormal expression of the Chemerin/ChemerinR1 axis is closely associated with the occurrence and development of various vascular inflammatory diseases. In hypertension, it promotes VSMC contraction and inflammatory cell infiltration, leading to elevated blood pressure and vascular function damage; in atherosclerosis, it promotes the recruitment and activation of inflammatory cells, exacerbates vascular wall inflammation, and leads to neointimal formation and vascular stenosis; it is also involved in inflammation in ischemia–reperfusion injury and post-angioplasty intimal hyperplasia; in obesity-related vascular diseases, it promotes adipose tissue inflammatory responses and vascular endothelial dysfunction; in RA, it exacerbates joint inflammation and damage; in ischemic stroke and CCH, it exerts protective or regulatory effects on neurons.

This axis has great potential for clinical transformation. Its expression can be used as a biomarker for various vascular inflammatory diseases, enabling early diagnosis, disease monitoring, and evaluation of treatment effects; intervening in this axis, whether by inhibiting its activity or activating it in specific cases, provides a new strategy to treat vascular inflammatory diseases. Drug development based on this axis is also actively ongoing. Although challenges, such as specificity, safety, and drug delivery, exist, they are expected to lead to the development of new drugs for clinical treatment.

## References

[B1] Abareshi A. Momenabadi S. Vafaei A. A. Bandegi A. R. Vakili A. (2021). Neuroprotective effects of chemerin on a mouse stroke model: behavioral and molecular dimensions. Neurochem. Res. 46 (12), 3301–3313. 10.1007/s11064-021-03432-9 34431027

[B2] Abu-Shakra M. Ahn S. S. Yoon T. Song J. J. Park Y.-B. Lee S. W. (2021). Serum adipokine profiles in patients with microscopic polyangiitis and granulomatosis with polyangiitis: an exploratory analysis. Plos One 16 (7), 1–14. 10.1371/journal.pone.0254226 34242326 PMC8270208

[B3] Albanesi C. Scarponi C. Pallotta S. Daniele R. Bosisio D. Madonna S. (2009). Chemerin expression marks early psoriatic skin lesions and correlates with plasmacytoid dendritic cell recruitment. J. Exp. Med. 206 (1), 249–258. 10.1084/jem.20080129 19114666 PMC2626680

[B4] Annalisa Del Prete L.M.-M. Mazzon C. Toffali L. Sozio F. Za L. Bosisio D. (2017). The atypical receptor CCRL2 is required for CXCR2-dependent neutrophil recruitment and tissue damage. Blood 130 (10), 1223–1234. 10.1182/blood-2017-04-777680 28743719

[B5] Arita M. Bianchini F. Aliberti J. Sher A. Chiang N. Hong S. (2005). Stereochemical assignment, antiinflammatory properties, and receptor for the omega-3 lipid mediator resolvin E1. J. Exp. Med. 201 (5), 713–722. 10.1084/jem.20042031 15753205 PMC2212834

[B6] Arita M. Ohira T. Sun Y. P. Elangovan S. Chiang N. Serhan C. N. (2007). Resolvin E1 selectively interacts with Leukotriene B4 receptor BLT1 and ChemR23 to regulate inflammation. J. Immunol. 178 (6), 3912–3917. 10.4049/jimmunol.178.6.3912 17339491

[B7] Arndt P. (2025). The role of chemerin in neutrophil activation and diseases of the lung. Biomedicines 13 (6), 1354. 10.3390/biomedicines13061354 40564073 PMC12189459

[B8] Aviv A. Levy D. (2012). Telomeres, atherosclerosis, and the hemothelium: the longer view. Annu. Rev. Med. 63 (1), 293–301. 10.1146/annurev-med-050311-104846 22017444 PMC4707036

[B9] Ballet R. LaJevic M. Huskey-Mullin N. Roach R. Brulois K. Huang Y. (2023). Chemerin triggers migration of a CD8 T cell subset with natural killer cell functions. Mol. Ther. 31 (10), 2887–2900. 10.1016/j.ymthe.2023.08.015 37641406 PMC10556222

[B10] Banas M. Zabieglo K. Kasetty G. Kapinska-Mrowiecka M. Borowczyk J. Drukala J. (2013). Chemerin is an antimicrobial agent in human epidermis. Plos One 8 (3), 8. 10.1371/journal.pone.0058709 23527010 PMC3604073

[B11] Bauer S. Wanninger J. Schmidhofer S. Weigert J. Neumeier M. Dorn C. (2011). Sterol regulatory element-binding protein 2 (SREBP2) activation after excess triglyceride storage induces chemerin in hypertrophic adipocytes. Endocrinology 152 (1), 26–35. 10.1210/en.2010-1157 21084441

[B12] Bauer S. Bala M. Kopp A. Eisinger K. Schmid A. Schneider S. (2012). Adipocyte chemerin release is induced by insulin without being translated to higher levels *in vivo* . Eur. J. Clin. Investigation 42 (11), 1213–1220. 10.1111/j.1365-2362.2012.02713.x 22924572

[B13] Ben Dhaou C. Del Prete A. Sozzani S. Parmentier M. (2021). CCRL2 modulates physiological and pathological angiogenesis during retinal development. Front. Cell Dev. Biol. 9, 808455. 10.3389/fcell.2021.808455 35004698 PMC8733553

[B14] Ben Dhaou C. Mandi K. Frye M. Acheampong A. Radi A. De Becker B. (2022). Chemerin regulates normal angiogenesis and hypoxia-driven neovascularization. Angiogenesis 25 (2), 159–179. 10.1007/s10456-021-09818-1 34524600 PMC9054887

[B15] Bennett M. R. Sinha S. Owens G. K. (2016). Vascular smooth muscle cells in atherosclerosis. Circulation Res. 118 (4), 692–702. 10.1161/CIRCRESAHA.115.306361 26892967 PMC4762053

[B16] Berg V. Sveinbjörnsson B. Bendiksen S. Brox J. Meknas K. Figenschau Y. (2010). Human articular chondrocytes express ChemR23 and chemerin; ChemR23 promotes inflammatory signalling upon binding the ligand chemerin21-157. Arthritis Res. and Ther. 12 (6), 12. 10.1186/ar3215 21192818 PMC3046541

[B17] Biasizzo M. Kopitar-Jerala N. (2020). Interplay between NLRP3 inflammasome and autophagy. Front. Immunol. 11, 591803. 10.3389/fimmu.2020.591803 33163006 PMC7583715

[B18] Bondue B. Wittamer V. Parmentier M. (2011). Chemerin and its receptors in leukocyte trafficking, inflammation and metabolism. Cytokine and Growth Factor Rev. 22 (5-6), 331–338. 10.1016/j.cytogfr.2011.11.004 22119008

[B19] Brian A. Zabel A. M. S. Eugene C. (2005). Butcher *chemokine-like receptor 1 expression and chemerin-directed chemotaxis distinguish plasmacytoid from Myeloid dendritic cells in human blood* , 174, 244–251. 10.4049/jimmunol.174.1.244 15611246

[B20] Buechler C. Feder S. Haberl E. M. Aslanidis C. (2019). Chemerin isoforms and activity in obesity. Int. J. Mol. Sci. 20 (5), 16. 10.3390/ijms20051128 30841637 PMC6429392

[B21] Carrión M. Frommer K. W. Pérez-García S. Müller-Ladner U. Gomariz R. P. Neumann E. (2019). The adipokine network in rheumatic joint diseases. Int. J. Mol. Sci. 20 (17), 1–30. 10.3390/ijms20174091 31443349 PMC6747092

[B22] Cash J. L. Hart R. Russ A. Dixon J. P. C. Colledge W. H. Doran J. (2008). Synthetic chemerin-derived peptides suppress inflammation through ChemR23. J. Exp. Med. 205 (4), 767–775. 10.1084/jem.20071601 18391062 PMC2292217

[B23] Cash J. Christian A. Greaves D. (2010). Chemerin peptides promote phagocytosis in a ChemR23-and syk-dependent manner. J. Immunol. 184 (9), 5315–5324. 10.4049/jimmunol.0903378 20363975 PMC4237835

[B24] Cash J. L. Bena S. Headland S. E. McArthur S. Brancaleone V. Perretti M. (2013). Chemerin15 inhibits neutrophil‐mediated vascular inflammation and myocardial ischemia‐reperfusion injury through ChemR23. EMBO Reports 14 (11), 999–1007. 10.1038/embor.2013.138 23999103 PMC3818079

[B25] Cash J. Bass M. D. Campbell J. Barnes M. Kubes P. Martin P. (2014). Resolution mediator Chemerin15 reprograms the wound microenvironment to promote repair and reduce scarring. Curr. Biol. 24 (12), 1406–1414. 10.1016/j.cub.2014.06.010 24881877 PMC4064685

[B26] Chen X. Lu J. Bao J. Guo J. Shi J. Wang Y. (2013). Adiponectin: a biomarker for rheumatoid arthritis? Cytokine and Growth Factor Rev. 24 (1), 83–89. 10.1016/j.cytogfr.2012.07.004 22910140

[B27] Chen S. Han C. Bian S. Chen J. Feng X. Li G. (2021). Chemerin-9 attenuates experimental abdominal aortic aneurysm formation in ApoE−/− mice. J. Oncol. 2021, 1–15. 10.1155/2021/6629204 33953746 PMC8068550

[B28] Chen Y. Liu Z. Gong P. Zhang H. Chen Y. Yao S. (2022). The Chemerin/CMKLR1 axis is involved in the recruitment of microglia to Aβ deposition through p38 MAPK pathway. Int. J. Mol. Sci. 23 (16), 19. 10.3390/ijms23169041 36012305 PMC9409288

[B29] Chen R. McVey D. G. Shen D. Huang X. Ye S. (2023). Phenotypic switching of vascular smooth muscle cells in atherosclerosis. J. Am. Heart Assoc. 12 (20), e031121. 10.1161/JAHA.123.031121 37815057 PMC10757534

[B30] Chen Y. Song Y. Wang Z. Lai Y. Yin W. Cai Q. (2024). The chemerin-CMKLR1 axis in keratinocytes impairs innate host defense against cutaneous *Staphylococcus aureus* infection. Cell. and Mol. Immunol. 21 (6), 533–545. 10.1038/s41423-024-01152-y 38532043 PMC11143357

[B31] Christos G Kostopoulos S. G. S. Varakis J. N. Apostolakis E. Papadaki H. H. (2014). Chemerin and CMKLR1 expression in human arteries and periadventitial fat: a possible role for local chemerin in atherosclerosis?. BMC Cardiovasc. Disord. 14, 1–9. 10.1186/1471-2261-14-56 24779513 PMC4022413

[B32] Conde J. Gomez R. Bianco G. Scotece M. Lear P. Dieguez C. (2011). Expanding the adipokine network in cartilage: identification and regulation of novel factors in human and murine chondrocytes. Ann. Rheumatic Dis. 70 (3), 551–559. 10.1136/ard.2010.132399 21216818

[B33] De Henau O. D. G.-N. Imbault V. Robert V. De Poorter C. Mcheik S. Galés C. (2016). Signaling properties of chemerin receptors CMKLR1, GPR1 and CCRL2, 11, 10. 10.1371/journal.pone.0164179 PMC5055294 27716822

[B34] De Palma G. Castellano G. Del Prete A. Sozzani S. Fiore N. Loverre A. (2011). The possible role of ChemR23/Chemerin axis in the recruitment of dendritic cells in lupus nephritis. Kidney Int. 79 (11), 1228–1235. 10.1038/ki.2011.32 21346723

[B35] de Poorter C. Baertsoen K. Lannoy V. Parmentier M. Springael J. Y. (2013). Consequences of ChemR23 heteromerization with the chemokine receptors CXCR4 and CCR7. Plos One 8 (2), 10. 10.1371/journal.pone.0058075 23469143 PMC3585228

[B36] Dimitriadis E. G. C. Kritikou E. Kouskouni E. (2018). Chemerin induces endothelial cell inflammation: activation of nuclear factor-kappa beta and monocyte-endothelial adhesion. Oncotarget 9 (30), 16678–16692. 10.18632/oncotarget.24659 29682177 PMC5908278

[B37] Dranse H. J. Muruganandan S. Fawcett J. P. Sinal C. J. (2016). Adipocyte-secreted chemerin is processed to a variety of isoforms and influences MMP3 and chemokine secretion through an NFkB-dependent mechanism. Mol. Cell. Endocrinol. 436, 114–129. 10.1016/j.mce.2016.07.017 27461525

[B38] Du X.-Y. Zabel B. A. Myles T. Allen S. J. Handel T. M. Lee P. P. (2009). Regulation of chemerin bioactivity by plasma carboxypeptidase N, carboxypeptidase B (activated thrombin-activable fibrinolysis inhibitor), and platelets. J. Biol. Chem. 284 (2), 751–758. 10.1074/jbc.M805000200 19010784 PMC2613638

[B39] Eichelmann F. Schulze M. B. Wittenbecher C. Menzel J. Weikert C. di Giuseppe R. (2019). Chemerin as a biomarker linking inflammation and cardiovascular diseases. J. Am. Coll. Cardiol. 73 (3), 378–379. 10.1016/j.jacc.2018.10.058 30678766

[B40] Evans B. R. Schulz J. Triantafyllidou V. Yerly A. Thakur M. Angliker N. (2025). ChemR23 prevents phenotypic switching of vascular smooth muscle cells into macrophage-like foam cells in atherosclerosis. Cardiovasc. Res. 122, 1–19. 10.1093/cvr/cvaf258 41264461 PMC13017563

[B41] Fabienne Mackay H. L. Dietrich S. Gehr G. Werner L. (1993). in Tumor necrosis factor α (TNF-α)-induced cell adhesion to human endothelial cells is under dominant control of one TNF receptor type, TNF-R55, 177, 1277–1286. 10.1084/jem.177.5.1277 PMC2190994 8386742

[B42] Ferland D. Darios E. S. Neubig R. R. Sjögren B. Truong N. Torres R. (2017). Chemerin-induced arterial contraction is Gi- and calcium-dependent. Vasc. Pharmacol. 88, 30–41. 10.1016/j.vph.2016.11.009 27890480 PMC5235970

[B43] Ferland D. Mullick A. Watts S. (2020). Chemerin as a driver of hypertension: a consideration. Am. J. Hypertens. 33 (11), 975–986. 10.1093/ajh/hpaa084 32453820 PMC7759724

[B44] Fernández-Friera L. Fuster V. López-Melgar B. Oliva B. Sánchez-González J. Macías A. (2019). Vascular inflammation in subclinical atherosclerosis detected by hybrid PET/MRI. J. Am. Coll. Cardiol. 73 (12), 1371–1382. 10.1016/j.jacc.2018.12.075 30922468

[B45] Gao C. Shi J. Zhang J. Li Y. Zhang Y. (2022). Chemerin promotes proliferation and migration of ovarian cancer cells by upregulating expression of PD-L1. J. Zhejiang University-Science B 23 (2), 164–170. 10.1631/jzus.B2100392 35187890 PMC8861558

[B46] Ge X. Y. Y. Zhao L. Bury L. Gresele P. Berube C. Leung L. L. (2018). Prochemerin cleavage by factor XIa links coagulation and inflammation . Blood 131 (3), 353–364. 10.1182/blood-2017-07-792580 29158361 PMC5774209

[B47] Goralski K. B. McCarthy T. C. Hanniman E. A. Zabel B. A. Butcher E. C. Parlee S. D. (2007). Chemerin, a novel adipokine that regulates adipogenesis and adipocyte metabolism. J. Biol. Chem. 282 (38), 28175–28188. 10.1074/jbc.M700793200 17635925

[B48] Gu P. Cheng M. Hui X. Lu B. Jiang W. Shi Z. (2015). Elevating circulation chemerin level is associated with endothelial dysfunction and early atherosclerotic changes in essential hypertensive patients. J. Hypertens. 33 (8), 1624–1632. 10.1097/HJH.0000000000000588 26136068

[B49] Gunawan S. Elger T. Loibl J. Fererberger T. Sommersberger S. Kandulski A. (2022). Urinary chemerin as a potential biomarker for inflammatory bowel disease. Front. Med. 9, 1058108. 10.3389/fmed.2022.1058108 36438059 PMC9691457

[B50] Guo X. Fu Y. Xu Y. Weng S. Liu D. Cui D. (2012). Chronic mild restraint stress rats decreased CMKLR1 expression in distinct brain region. Neurosci. Lett. 524 (1), 25–29. 10.1016/j.neulet.2012.06.075 22796467

[B51] Hamlett E. D. Hjorth E. Ledreux A. Gilmore A. Schultzberg M. Granholm A. C. (2020). RvE1 treatment prevents memory loss and neuroinflammation in the Ts65Dn mouse model of Down syndrome. Glia 68 (7), 1347–1360. 10.1002/glia.23779 31944407 PMC7205572

[B52] Hansson G. K. Hermansson A. (2011). The immune system in atherosclerosis. Nat. Immunol. 12 (3), 204–212. 10.1038/ni.2001 21321594

[B53] He D. Kou X. Luo Q. Yang R. Liu D. Wang X. (2015). Enhanced M1/M2 macrophage ratio promotes orthodontic root resorption. J. Dent. Res. 94 (1), 129–139. 10.1177/0022034514553817 25344334

[B54] Helfer G. Wu Q. (2018). Chemerin: a multifaceted adipokine involved in metabolic disorders. J. Endocrinol. 238 (2), R79–R94. 10.1530/JOE-18-0174 29848608 PMC6026924

[B55] Helfer G. Ross A. W. Thomson L. M. Mayer C. D. Stoney P. N. McCaffery P. J. (2016). A neuroendocrine role for chemerin in hypothalamic remodelling and photoperiodic control of energy balance. Sci. Rep. 6, 12. 10.1038/srep26830 27225311 PMC4880918

[B56] Herová M. Schmid M. Gemperle C. Hersberger M. (2015). ChemR23, the receptor for chemerin and resolvin E1, is expressed and functional on M1 but not on M2 macrophages. J. Immunol. 194 (5), 2330–2337. 10.4049/jimmunol.1402166 25637017

[B57] Hsu M. K. H. Qiao L. Ho V. Zhang B. H. Zhang H. Teoh N. (2006). Ethanol reduces p38 kinase activation and cyclin D1 protein expression after partial hepatectomy in rats. J. Hepatology 44 (2), 375–382. 10.1016/j.jhep.2005.07.031 16226824

[B58] Hu S. Zhang C. Huang Z. Zhao D. Wang Y. Li J. (2020). Chemerin facilitates intervertebral disc degeneration via TLR4 and CMKLR1 and activation of NF-kB signaling pathway . Aging (Albany NY) 12 (11), 103339. 10.18632/aging.103339 PMC7343479 32526705

[B59] Huang H. Tong T. T. Yau L. F. Wang J. R. Lai M. H. Zhang C. R. (2020). Chemerin isoform analysis in human biofluids using an LC/MRM-MS-based targeted proteomics approach with stable isotope-labeled standard. Anal. Chim. Acta 1139, 79–87. 10.1016/j.aca.2020.08.062 33190712

[B60] Huang Y. Li S. Yang Y. Li K. Wen L. Li J. (2024). Chemerin 15 peptide reduces neuroinflammation *via* the ChemR23 receptor after ischemia-reperfusion injury. Neural Regen. Res. 20, 1–14. 10.4103/NRR.NRR-D-24-00137 39248165 PMC13378918

[B61] Imiela A. M. Stępnicki J. Zawadzka P. S. Bursa A. Pruszczyk P. (2025). Chemerin as a driver of cardiovascular diseases: new perspectives and future directions. Biomedicines 13 (6), 1481. 10.3390/biomedicines13061481 40564199 PMC12190777

[B62] Jaworek J. Szklarczyk J. Kot M. Góralska M. Jaworek A. Bonior J. (2019). Chemerin alleviates acute pancreatitis in the rat thorough modulation of NF-κB signal. Pancreatology 19 (3), 401–408. 10.1016/j.pan.2019.02.005 30833212

[B63] Ji J. Zhong H. Wang Y. Liu J. Tang J. Liu Z. (2024). Chemerin attracts neutrophil reverse migration by interacting with C-C motif chemokine receptor-like 2. Cell Death and Dis. 15 (6), 10. 10.1038/s41419-024-06820-5 38890311 PMC11189533

[B64] Jiang Y. Liu P. Jiao W. Meng J. Feng J. (2017). Gax suppresses chemerin/CMKLR1‐induced preadipocyte biofunctions through the inhibition of Akt/mTOR and ERK signaling pathways. J. Cell. Physiology 233 (1), 572–586. 10.1002/jcp.25918 28326537

[B65] Johnson J. L. (2017). Metalloproteinases in atherosclerosis. Eur. J. Pharmacol. 816, 93–106. 10.1016/j.ejphar.2017.09.007 28893577

[B66] Jourd'heuil D. Xiong W. Luo Y. Wu L. Liu F. Liu H. (2016). Chemerin stimulates vascular smooth muscle cell proliferation and carotid neointimal hyperplasia by activating mitogen-activated protein kinase signaling. Plos One 11 (10), 1–14. 10.1371/journal.pone.0165305 27792753 PMC5085037

[B68] Kaneko Y. O. S. Ohshima S. Takeuchi T. (2011). Chemerin activates fibroblast-like synoviocytes in patients with rheumatoid arthritis. Arthritis Rheum. 63 (11), 3410–3420. 10.1186/ar3475 PMC3308089 21959042

[B69] Kantarci A. Aytan N. Palaska I. Stephens D. Crabtree L. Benincasa C. (2018). Combined administration of resolvin E1 and lipoxin A4 resolves inflammation in a murine model of Alzheimer's disease. Exp. Neurol. 300, 111–120. 10.1016/j.expneurol.2017.11.005 29126887

[B70] Kaur J. Adya R. Tan B. K. Chen J. Randeva H. S. (2010). Identification of chemerin receptor (ChemR23) in human endothelial cells: Chemerin-induced endothelial angiogenesis. Biochem. Biophysical Res. Commun. 391 (4), 1762–1768. 10.1016/j.bbrc.2009.12.150 20044979

[B71] Kennedy A. J. Davenport A. P. (2018). International union of basic and clinical pharmacology CIII: chemerin receptors CMKLR1 (Chemerin(1)) and GPR1 (Chemerin(2)) nomenclature, pharmacology, and function. Pharmacol. Rev. 70 (1), 174–196. 10.1124/pr.116.013177 29279348 PMC5744648

[B72] Kim M. Lee J. (2024). Molecular foundations of inflammatory diseases: insights into inflammation and inflammasomes. Curr. Issues Mol. Biol. 46 (1), 469–484. 10.3390/cimb46010030 38248332 PMC10813887

[B73] Kim H. Lee J. H. Lee S. K. Song N. Y. Son S. H. Kim K. R. (2020). Chemerin treatment inhibits the growth and bone invasion of breast cancer cells. Int. J. Mol. Sci. 21 (8), 1–19. 10.3390/ijms21082871 32325994 PMC7216174

[B74] Kim I. Park H. Hwang I. Moon D. Yun H. Lee E. J. (2021). Discovery of chemerin as the new chemoattractant of human mesenchymal stem cells. Cell and Biosci. 11 (1), 120. 10.1186/s13578-021-00631-3 34210352 PMC8252297

[B75] Kiymet Bozaoglu J. E. C. Stocker C. J. Zaibi M. S. Segal D. Konstantopoulos N. Morrison S. (2010). Chemerin, A novel adipokine in the regulation of angiogenesis. 10.1210/jc.2010-0042 PMC2869547 20237162

[B76] Krautbauer S. Wanninger J. Eisinger K. Hader Y. Beck M. Kopp A. (2013). Chemerin is highly expressed in hepatocytes and is induced in non-alcoholic steatohepatitis liver. Exp. Mol. Pathology 95 (2), 199–205. 10.1016/j.yexmp.2013.07.009 23906870

[B77] Kulig P. Kantyka T. Zabel B. A. Banas M. Chyra A. Stefanska A. (2011). Regulation of chemerin chemoattractant and antibacterial activity by Human Cysteine cathepsins. J. Immunol. 187 (3), 1403–1410. 10.4049/jimmunol.1002352 21715684 PMC3140563

[B78] Kunimoto H. Kazama K. Takai M. Oda M. Okada M. Yamawaki H. (2015). Chemerin promotes the proliferation and migration of vascular smooth muscle and increases mouse blood pressure. Am. J. Physiology-Heart Circulatory Physiology 309 (5), H1017–H1028. 10.1152/ajpheart.00820.2014 26254337

[B79] Lee J.-M. Lee J.-H Song M.-K. Kim Y.-J (2021). NXP031 improves cognitive impairment in a chronic cerebral hypoperfusion-induced vascular dementia rat model through Nrf2 signaling. Int. J. Mol. Sci. 22 (12), 6285. 10.3390/ijms22126285 34208092 PMC8230952

[B80] Lei Q. Yi T. Chen C. (2018). NF-κB-Gasdermin D (GSDMD) axis couples oxidative stress and NACHT, LRR and PYD domains-containing protein 3 (NLRP3) inflammasome-mediated cardiomyocyte pyroptosis following myocardial infarction. Med. Sci. Monit. 24, 6044–6052. 10.12659/MSM.908529 30161099 PMC6128186

[B81] Lei Z. Lu Y. Bai X. Jiang Z. Yu Q. (2020). *Chemerin-9 peptide enhances memory and ameliorates Aβ* _1–42_ *-Induced object memory impairment in mice* . Biol. Pharm. Bull. 43 (2), 272–283. 10.1248/bpb.b19-00510 31748466

[B82] Lensing M. Jabbari A. (2022). An overview of JAK/STAT pathways and JAK inhibition in alopecia areata. Front. Immunol. 13, 955035. 10.3389/fimmu.2022.955035 36110853 PMC9470217

[B83] Li Y. Z. X. Wang Y. Liu X. Chen L. Zhao J. (2021). Chemerin/ChemR23 regulates cementoblast function and tooth resorption in mice *via* inflammatory factors. J. Periodontol. 92 (10), 1470–1482. 10.1002/JPER.20-0675 33289084

[B84] Liberale L. Badimon L. Montecucco F. Lüscher T. F. Libby P. Camici G. G. (2022). Inflammation, aging, and cardiovascular disease. J. Am. Coll. Cardiol. 79 (8), 837–847. 10.1016/j.jacc.2021.12.017 35210039 PMC8881676

[B85] Lin Y. Yang X. Yue W. Xu X. Li B. Zou L. (2014). Chemerin aggravates DSS-induced colitis by suppressing M2 macrophage polarization. Cell. and Mol. Immunol. 11 (4), 355–366. 10.1038/cmi.2014.15 24727542 PMC4085517

[B86] Lin Y. Yang X. Liu W. Li B. Yin W. Shi Y. (2017). Chemerin has a protective role in hepatocellular carcinoma by inhibiting the expression of IL-6 and GM-CSF and MDSC accumulation. Oncogene 36 (25), 3599–3608. 10.1038/onc.2016.516 28166197

[B87] Lin N. Song X. Chen B. Ye H. Wang Y. Cheng X. (2020). Leptin is upregulated in epididymitis and promotes apoptosis and IL-1β production in epididymal epithelial cells by activating the NLRP3 inflammasome. Int. Immunopharmacol. 88, 9. 10.1016/j.intimp.2020.106901 33182054

[B88] Lin X. Zhao L. Cai H. Chang X. Tang Y. Luo T. (2025). Structural insights into the distinct ligand recognition and signaling of the chemerin receptors CMKLR1 and GPR1. Protein and Cell 16 (5), 381–385. 10.1093/procel/pwae073 39752296 PMC12120241

[B89] Lisa Patel S. J. C. Chambers J. K. Macphee C. H. Macphee C. H. (2001). Expression and functional analysis of chemokine receptors in human peripheral blood leukocyte populations. Cytokine 14 (1), 27–36. 10.1006/cyto.2000.0851 11298490

[B90] Liu G. Liu Q. Shen Y. Kong D. Gong Y. Tao B. (2018). Early treatment with resolvin E1 facilitates myocardial recovery from ischaemia in mice. Br. J. Pharmacol. 175 (8), 1205–1216. 10.1111/bph.14041 28925017 PMC5866979

[B91] Liu H. Xiong W. Luo Y. Chen H. He Y. Cao Y. (2019). Adipokine chemerin stimulates progression of atherosclerosis in ApoE−/− mice. BioMed Res. Int. 2019, 1–9. 10.1155/2019/7157865 31781638 PMC6875193

[B92] Liu C. Chen Y. Cui W. Cao Y. Zhao L. Wang H. (2021). Inhibition of neuronal necroptosis mediated by RIP1/RIP3/MLKL provides neuroprotective effects on kaolin-induced hydrocephalus in mice. Cell Prolif. 54 (9), 14. 10.1111/cpr.13108 34374150 PMC8450124

[B93] Liu L. Zhang J. Lu K. Zhang Y. Xu X. Deng J. (2024). ChemR23 signaling ameliorates brain injury *via* inhibiting NLRP3 inflammasome-mediated neuronal pyroptosis in ischemic stroke. J. Transl. Med. 22 (1), 23. 10.1186/s12967-023-04813-0 38178174 PMC10768115

[B94] Lu T. Li H. Zhou Y. Wei W. Ding L. Zhan Z. (2022). Neuroprotective effects of alisol A 24-acetate on cerebral ischaemia-reperfusion injury are mediated by regulating the PI3K/AKT pathway. J. Neuroinflammation 19 (1), 15. 10.1186/s12974-022-02392-3 35130910 PMC8822821

[B95] Lu Z. Liu J. Wan Q. Wu Y. Wu W. Chen Y. (2024). Chemerin promotes invasion of oral squamous cell carcinoma by stimulating IL-6 and TNF-alpha production via STAT3 activation. Mol. Biol. Rep. 51 (1), 436. 10.1007/s11033-024-09359-y 38520551

[B96] Luangsay S. Wittamer V. Bondue B. De Henau O. Rouger L. Brait M. (2009). Mouse ChemR23 is expressed in dendritic cell subsets and macrophages, and mediates an anti-inflammatory activity of chemerin in a lung disease model. J. Immunol. 183 (10), 6489–6499. 10.4049/jimmunol.0901037 19841182

[B97] Ma D. Li Q. Zhang H. (2025). Vascular smooth muscle cell dysfunction in cerebral small vessel disease: mechanisms and therapeutic potential. Cerebrovasc. Dis. 55 (1), 114–124. 10.1159/000545796 40209682

[B98] Man A. W. C. Zhou Y. Reifenberg G. Camp A. Münzel T. Daiber A. (2023). Deletion of adipocyte NOS3 potentiates high-fat diet-induced hypertension and vascular remodelling *via* chemerin. Cardiovasc. Res. 119 (17), 2755–2769. 10.1093/cvr/cvad164 37897505 PMC10757584

[B99] Mariani F. Roncucci L. (2015). Chemerin/chemR23 axis in inflammation onset and resolution. Inflamm. Res. 64 (2), 85–95. 10.1007/s00011-014-0792-7 25548799

[B100] Matsuyama H. Shindo A. Shimada T. Yata K. Wakita H. Takahashi R. (2020). Chronic cerebral hypoperfusion activates AIM2 and NLRP3 inflammasome. Brain Res. 1736, 8. 10.1016/j.brainres.2020.146779 32171704

[B101] McMillan R. Kirabo A. (2025). Chemerin as a mediator of hypertension and cardiometabolic diseases (A comprehensive review). Curr. Hypertens. Rep. 28 (1), 4. 10.1007/s11906-025-01354-3 41457200 PMC12745319

[B102] Migeotte I. Goriely S. Willems F. Parmentier M. Parmentier M. (2002). Distribution and regulation of expression of the putative human chemokine receptor HCR in leukocyte populations. Eur. J. Immunol. 32, 494–501. 10.1002/1521-4141(200202)32:2<494::AID-IMMU494>3.0.CO;2-Y 11828366

[B103] Mitsis A. Khattab E. Myrianthefs M. Tzikas S. Kadoglou N. P. E. Fragakis N. (2024). Chemerin in the spotlight: revealing its multifaceted role in acute myocardial infarction. Biomedicines 12 (9), 2133. 10.3390/biomedicines12092133 39335646 PMC11428948

[B67] Monnier J. S. O'Hara E. Huang K. Tu H. Butcher E. C. Zabel B. A. (2001). Expression, regulation, and function of atypical chemerin receptor CCRL2 on endothelial cells. J. Immunol. 189 (2), 956–967. 10.4049/jimmunol.1102871 PMC3428203 22696441

[B104] Nam M.-H. Son W. R. Lee Y. S. Lee K. W. (2016). Glycolaldehyde-derived advanced glycation end products (glycol-AGEs)-induced vascular smooth muscle cell dysfunction is regulated by the AGES-receptor (RAGE) axis in endothelium. Cell Commun. and Adhesion 22 (2-6), 67–78. 10.1080/15419061.2016.1225196 27602595

[B105] Neves K. B. Nguyen Dinh Cat A. Lopes R. A. M. Rios F. J. Anagnostopoulou A. Lobato N. S. (2015). Chemerin regulates crosstalk between adipocytes and vascular cells through nox. Hypertension 66 (3), 657–666. 10.1161/HYPERTENSIONAHA.115.05616 26150435

[B106] Özcan E. Saygun N. I. Serdar M. A. Kurt N. (2015). Evaluation of the salivary levels of visfatin, chemerin, and progranulin in periodontal inflammation. Clin. Oral Investig. 19 (4), 921–928. 10.1007/s00784-014-1308-0 25164155

[B107] Parolini S. Santoro A. Marcenaro E. Luini W. Massardi L. Facchetti F. (2007). The role of chemerin in the colocalization of NK and dendritic cell subsets into inflamed tissues. Blood 109 (9), 3625–3632. 10.1182/blood-2006-08-038844 17202316

[B108] Peng L. Yu Y. Liu J. Li S. He H. Cheng N. (2015). The chemerin receptor CMKLR1 is a functional receptor for Amyloid-β peptide. J. Alzheimers Dis. 43 (1), 227–242. 10.3233/JAD-141227 25079809

[B109] Poh L. Sim W. L. Jo D. G. Dinh Q. N. Drummond G. R. Sobey C. G. (2022). The role of inflammasomes in vascular cognitive impairment. Mol. Neurodegener. 17 (1), 28. 10.1186/s13024-021-00506-8 35000611 PMC8744307

[B110] Rennier K. Shin W. J. Krug E. Virdi G. Pachynski R. K. (2020). Chemerin reactivates PTEN and suppresses PD-L1 in tumor cells *via* modulation of a novel CMKLR1-mediated signaling Cascade. Clin. Cancer Res. 26 (18), 5019–5035. 10.1158/1078-0432.CCR-19-4245 32605911

[B111] Rey C. Nadjar A. Buaud B. Vaysse C. Aubert A. Pallet V. (2016). Resolvin D1 and E1 promote resolution of inflammation in microglial cells *in vitro* . Brain Behav. Immun. 55, 249–259. 10.1016/j.bbi.2015.12.013 26718448

[B112] Rosales C. Demaurex N. Lowell C. A. Uribe-Querol E. (2016). Neutrophils: their role in innate and adaptive immunity. J. Immunol. Res. 2016, 1–2. 10.1155/2016/1469780 27006954 PMC4783580

[B113] Rourke J. L. M. S. Dranse H. J. McMullen N. M. Sinal C. J. (2014). Gpr1 is an active chemerin receptor influencing glucose homeostasis in obese mice. J. Endocrinol. 222 (2), 201–215. 10.1530/joe-14-0069 24895415

[B114] Sato K. Yoshizawa H. Seki T. Shirai R. Yamashita T. Okano T. (2019). Chemerin-9, a potent agonist of chemerin receptor (ChemR23), prevents atherogenesis. Clin. Sci. 133 (16), 1779–1796. 10.1042/CS20190336 31399499

[B115] Segal D. Bozaoglu K. Bolton K. McMillan J. Zimmet P. Jowett J. (2007). Chemerin is a novel adipokine associated with obesity and metabolic syndrome. Endocrinology 148 (10), 4687–4694. 10.1210/en.2007-0175 17640997

[B116] Serhan C. N. (2007). Resolution phase of inflammation: novel endogenous anti-inflammatory and proresolving lipid mediators and pathways. Annu. Rev. Immunol. 25 (1), 101–137. 10.1146/annurev.immunol.25.022106.141647 17090225

[B117] Serhan C. (2014). Pro-resolving lipid mediators are leads for resolution physiology. Nature 510 (7503), 92–101. 10.1038/nature13479 24899309 PMC4263681

[B118] Shang J. Wang L. Zhang Y. Zhang S. Ning L. Zhao J. (2019). Chemerin/ChemR23 axis promotes inflammation of glomerular endothelial cells in diabetic nephropathy. J. Cell. Mol. Med. 23 (5), 3417–3428. 10.1111/jcmm.14237 30784180 PMC6484295

[B119] Sigfridsson E. Marangoni M. Hardingham G. E. Horsburgh K. Fowler J. H. (2020). Deficiency of Nrf2 exacerbates white matter damage and microglia/macrophage levels in a mouse model of vascular cognitive impairment. J. Neuroinflammation 17 (1), 15. 10.1186/s12974-020-02038-2 33261626 PMC7709339

[B120] Song Y. Y. W. Dan Y. Sheng J. Chen Y. Wang Z. Lai Y. (2022). *Epithelial chemerin–CMKLR1 signaling restricts microbiota* driven colonic neutrophilia and tumorigenesis by up-regulating *lactoperoxidase* . Proc. Natl. Acad. Sci. U. S. A. 119 (29), e2205574119. 10.1073/pnas.2205574119 35858331 PMC9304024

[B121] Stojek M. (2017). The role of chemerin in human disease. Postepy Hig. I Med. Doswiadczalnej 71, 110–117. 10.5604/01.3001.0010.3795 28258671

[B122] Su S. Wang Y. Q. Wu Y. F. Wang D. P. Lin Q. Hai J. (2016). Cannabinoid receptor agonist WIN55,212-2 and fatty acid amide hydrolase inhibitor URB597 may protect against.cognitive impairment in rats of chronic cerebral hypoperfusion *via* PI3K/AKT signaling. Behav. Brain Res. 313, 334–344. 10.1016/j.bbr.2016.07.009 27424778

[B123] Su X. Cheng Y. Zhang G. Wang B. (2021). Chemerin in inflammatory diseases. Clin. Chim. Acta 517, 41–47. 10.1016/j.cca.2021.02.010 33631197

[B124] Sulicka-Grodzicka J. Surdacki A. Surmiak M. Sanak M. Wizner B. Sydor W. (2022). Chemerin as a potential marker of resolution of inflammation in COVID-19 infection. Biomedicines 10 (10), 14. 10.3390/biomedicines10102462 36289725 PMC9599036

[B125] Sun R. Peng M. Xu P. Huang F. Xie Y. Li J. (2020). Low-density lipoprotein receptor (LDLR) regulates NLRP3-mediated neuronal pyroptosis following cerebral ischemia/reperfusion injury. J. Neuroinflammation 17 (1), 17. 10.1186/s12974-020-01988-x 33153475 PMC7643474

[B126] Sun J.-X. Zhang C. Cheng Z. B. Tang M. Y. Liu Y. Z. Jiang J. F. (2021). Chemerin in atherosclerosis. Clin. Chim. Acta 520, 8–15. 10.1016/j.cca.2021.05.015 34022243

[B127] Suzuki H. Otsuka T. Hitora-Imamura N. Ishimura K. Fukuda H. Fujiwara K. (2021). Resolvin E1 attenuates chronic pain-induced depression-like behavior in mice: possible involvement of chemerin receptor ChemR23. Biol. and Pharm. Bull. 44 (10), 1548–1550. 10.1248/bpb.b21-00461 34602564

[B128] Tan L. Lu X. Danser A. H. J. Verdonk K. (2023). The role of chemerin in metabolic and cardiovascular disease: a literature review of its physiology and pathology from a nutritional perspective. Nutrients 15 (13), 2878. 10.3390/nu15132878 37447205 PMC10343651

[B129] Tang C. Chen G. Wu F. Cao Y. Yang F. You T. (2023). Endothelial CCRL2 induced by disturbed flow promotes atherosclerosis *via* chemerin-dependent β2 integrin activation in monocytes. Cardiovasc. Res. 119 (9), 1811–1824. 10.1093/cvr/cvad085 37279540 PMC10405567

[B130] Tang S. Jiang Y. Wu Y. Su X. Wang S. Chen R. (2026). Impact of severe infections on the risk of acute cardiovascular and cerebrovascular diseases: a prospective cohort study. Am. J. Prev. Cardiol. 26, 101436. 10.1016/j.ajpc.2026.101436 41630960 PMC12860620

[B131] Thiam H. R. Wong S. L. Wagner D. D. Waterman C. M. (2020). Cellular mechanisms of NETosis. Annu. Rev. Cell Dev. Biol. 36 (1), 191–218. 10.1146/annurev-cellbio-020520-111016 32663035 PMC8499668

[B132] Tiberio M. Parolini F. Sozzani S. Del Prete A. (2023). CCRL2 expression by specialized lung capillary endothelial cells controls NK cell homing in lung cancer. Cancer Immunol. Res. 11 (6), 924–938. 10.1158/2326-6066.CIR-22-0951 37343073

[B133] Tokizawa S S. N. Liu H.-Y. Fang D.-Y. Haraguchi Y. Oite T. Hoshino H. (2000). Infection of mesangial cells with HIV and SIV: identification of GPR1 as a coreceptor. Kidney Int. 58, 607–617. 10.1046/j.1523-1755.2000.00207.x 10916084

[B134] Treeck O. Buechler C. Ortmann O. (2019). Chemerin and cancer. Int. J. Mol. Sci. 20 (15), 1–16. 10.3390/ijms20153750 31370263 PMC6695761

[B135] van der Vorst E. P. C. Mandl M. Müller M. Neideck C. Jansen Y. Hristov M. (2019). Hematopoietic ChemR23 (Chemerin receptor 23) fuels atherosclerosis by sustaining an M1 macrophage-phenotype and guidance of plasmacytoid dendritic cells to murine lesions—brief report. Arteriosclerosis, Thrombosis, Vasc. Biol. 39 (4), 685–693. 10.1161/ATVBAHA.119.312386 30786742

[B136] Vermi W. Riboldi E. Wittamer V. Gentili F. Luini W. Marrelli S. (2005). Role of ChemR23 in directing the migration of myeloid and plasmacytoid dendritic cells to lymphoid organs and inflamed skin. J. Exp. Med. 201 (4), 509–515. 10.1084/jem.20041310 15728234 PMC2213064

[B137] Voet S. Srinivasan S. Lamkanfi M. van Loo G. (2019). Inflammasomes in neuroinflammatory and neurodegenerative diseases. EMBO Mol. Med. 11 (6), 1–16. 10.15252/emmm.201810248 31015277 PMC6554670

[B138] Waluga M. Hartleb M. Boryczka G. Kukla M. Zwirska-Korczala K. (2014). Serum adipokines in inflammatory bowel disease. World J. Gastroenterology 20 (22), 6912–6917. 10.3748/wjg.v20.i22.6912 24944482 PMC4051931

[B139] Wang Y. He H. Liu Z. J. Cao Z. G. Wang X. Y. Yang K. (2015). Effects of TNF-α on cementoblast differentiation, mineralization, and apoptosis. J. Dent. Res. 94 (9), 1225–1232. 10.1177/0022034515590349 26088424

[B140] Wang C. Xu Y. Wang X. Guo C. Wang T. Wang Z. Y. (2019). Dl-3-n-Butylphthalide inhibits NLRP3 inflammasome and mitigates alzheimer's-like pathology *via* Nrf2-TXNIP-TrX axis. Antioxidants and Redox Signal. 30 (11), 1411–1431. 10.1089/ars.2017.7440 29634349

[B141] Watts S. W. Dorrance A. M. Penfold M. E. Rourke J. L. Sinal C. J. Seitz B. (2013). Chemerin connects fat to arterial contraction. Arteriosclerosis, Thrombosis, Vasc. Biol. 33 (6), 1320–1328. 10.1161/ATVBAHA.113.301476 23559624 PMC3752465

[B142] Watts S. W. Darios E. S. Mullick A. E. Garver H. Saunders T. L. Hughes E. D. (2018). The chemerin knockout rat reveals chemerin dependence in female, but not male, experimental hypertension. FASEB J. 32 (12), 6596–6614. 10.1096/fj.201800479 29906243 PMC6219827

[B143] Weigert J. Obermeier F. Neumeier M. Wanninger J. Filarsky M. Bauer S. (2010). Circulating levels of chemerin and adiponectin are higher in ulcerative colitis and chemerin is elevated in Crohnʼs disease. Inflamm. Bowel Dis. 16 (4), 630–637. 10.1002/ibd.21091 19714754

[B144] Wen L. Qiu H. Li S. Huang Y. Tu Q. Lyu N. (2024). Vascular stent with immobilized anti-inflammatory chemerin 15 peptides mitigates neointimal hyperplasia and accelerates vascular healing. Acta Biomater. 179, 371–384. 10.1016/j.actbio.2024.02.022 38382829

[B145] Wittamer V. r. Franssen J. D. Vulcano M. Mirjolet J. F. Le Poul E. Migeotte I. (2003). Specific recruitment of antigen-presenting cells by Chemerin, a novel processed ligand from human inflammatory fluids. J. Exp. Med. 198 (7), 977–985. 10.1084/jem.20030382 14530373 PMC2194212

[B146] Wittamer V. Grégoire F. Robberecht P. Vassart G. Communi D. Parmentier M. (2004). The C-terminal nonapeptide of mature chemerin activates the chemerin receptor with low nanomolar potency. J. Biol. Chem. 279 (11), 9956–9962. 10.1074/jbc.M313016200 14701797

[B147] Wu D. Jin Y. Xing Y. Guo C. Tam L. S. (2023). Global, regional, and national incidence of six major immune-mediated inflammatory diseases: findings from the global burden of disease study 2019. eClinicalMedicine 64, 102193. 10.1016/j.eclinm.2023.102193 37731935 PMC10507198

[B148] Xie Y. Liu L. (2022). Role of Chemerin/ChemR23 axis as an emerging therapeutic perspective on obesity-related vascular dysfunction. J. Transl. Med. 20 (1), 15. 10.1186/s12967-021-03220-7 35317838 PMC8939091

[B149] Xu L.-L. Wu Y. F. Yan F. Li C. C. Dai Z. You Q. D. (2019). 5-(3,4-Difluorophenyl)-3-(6-methylpyridin-3-yl)-1,2,4-oxadiazole (DDO-7263), a novel Nrf2 activator targeting brain tissue, protects against MPTP-induced subacute Parkinson's disease in mice by inhibiting the NLRP3 inflammasome and protects PC12 cells against oxidative stress. Free Radic. Biol. Med. 134, 288–303. 10.1016/j.freeradbiomed.2019.01.003 30615919

[B150] Yang J. Ma X. J. Li L. Wang L. Chen Y. G. Liu J. (2017). Berberine ameliorates non-alcoholic steatohepatitis in ApoE-/- mice. Exp. Ther. Med. 14 (5), 4134–4140. 10.3892/etm.2017.5051 29075339 PMC5647746

[B151] Yang Y. Song J. Liu N. Wei G. Liu S. Zhang S. (2022). Salvianolic acid A relieves cognitive disorder after chronic cerebral ischemia: involvement of Drd2/Cryab/NF-κB pathway. Pharmacol. Res. 175, 13. 10.1016/j.phrs.2021.105989 34800628

[B152] Ye J. Yan H. M. Xia M. F. Wang Y. Chang X. X. Yao X. Z. (2015). Efficacy of berberine in patients with non-alcoholic fatty liver disease. Plos One 10 (8), 1–16. 10.1371/journal.pone.0134172 26252777 PMC4529214

[B153] Yoshimura T. Oppenheim J. J. (2008). Chemerin reveals its chimeric nature. J. Exp. Med. 205 (10), 2187–2190. 10.1084/jem.20081736 18809717 PMC2556799

[B154] Yu M. Yang Y. Huang C. Ge L. Xue L. Xiao Z. (2022). Chemerin: a functional adipokine in reproductive health and diseases. Biomedicines 10 (8), 1910. 10.3390/biomedicines10081910 36009457 PMC9406010

[B155] Yukihiko Momiyama N. I. Adachi H. Saita E. Fairweather D. L. (2014). Inflammation, atherosclerosis and coronary artery disease. Clin. Med. Insights Cardiol. 8, 67–70. 10.4137/CMC.S39423 27594791 PMC5003124

[B156] Zabel B. A. Allen S. J. Kulig P. Allen J. A. Cichy J. Handel T. M. (2005). Chemerin activation by serine proteases of the coagulation, fibrinolytic, and inflammatory cascades. J. Biol. Chem. 280 (41), 34661–34666. 10.1074/jbc.M504868200 16096270

[B157] Zabel B. A. Ohyama T. Zuniga L. Kim J. Y. Johnston B. Allen S. J. (2006). Chemokine-like receptor 1 expression by macrophages *in vivo:* regulation by TGF-β and TLR ligands. Exp. Hematol. 34 (8), 1106–1114. 10.1016/j.exphem.2006.03.011 16863918

[B158] Zabel B. A. Nakae S. Zúñiga L. Kim J. Y. Ohyama T. Alt C. (2008). Mast cell–expressed orphan receptor CCRL2 binds chemerin and is required for optimal induction of IgE-mediated passive cutaneous anaphylaxis. J. Exp. Med. 205 (10), 2207–2220. 10.1084/jem.20080300 18794339 PMC2556791

[B159] Zhang J. Zhang Y. Liu L. Zhang M. Zhang X. Deng J. (2025). Chemerin-9 is neuroprotective in APP/PS1 transgenic mice by inhibiting NLRP3 inflammasome and promoting microglial clearance of Aβ. J. Neuroinflammation 22 (1), 5. 10.1186/s12974-024-03325-y 39780188 PMC11716275

[B160] Zhang Y. Xu N. Ding Y. Zhang Y. Li Q. Flores J. (2018). Chemerin suppresses neuroinflammation and improves neurological recovery *via* CaMKK2/AMPK/Nrf2 pathway after germinal matrix hemorrhage in neonatal rats. Brain, Behav. Immun. 70, 179–193. 10.1016/j.bbi.2018.02.015 29499303 PMC5953818

[B161] Zhang Y. Xu N. Ding Y. Doycheva D. M. Zhang Y. Li Q. (2019). Chemerin reverses neurological impairments and ameliorates neuronal apoptosis through ChemR23/CAMKK2/AMPK pathway in neonatal hypoxic-ischemic encephalopathy. Cell Death and Dis. 10, 15. 10.1038/s41419-019-1374-y 30718467 PMC6362229

[B162] Zhang J. Yin Z. Xu Y. Wei C. Peng S. Zhao M. (2023). Resolvin E1/ChemR23 protects against hypertension and vascular remodeling in angiotensin II–Induced hypertensive mice. Hypertension 80 (12), 2650–2664. 10.1161/HYPERTENSIONAHA.123.21348 37800344

[B163] Zhang Y. Zhang J. Zhao Y. Zhang Y. Liu L. Xu X. (2023). ChemR23 activation attenuates cognitive impairment in chronic cerebral hypoperfusion by inhibiting NLRP3 inflammasome-induced neuronal pyroptosis. Cell Death and Dis. 14 (11), 721. 10.1038/s41419-023-06237-6 37932279 PMC10628255

[B164] Zhao L. Yang W. Yang X. Lin Y. Lv J. Dou X. (2014). Chemerin suppresses murine allergic asthma by inhibiting CCL2 production and subsequent airway recruitment of inflammatory dendritic cells. Allergy 69 (6), 763–774. 10.1111/all.12408 24758146

